# Targeting METTL3 enhances the chemosensitivity of non-small cell lung cancer cells by decreasing ABCC2 expression in an m^6^A-YTHDF1-dependent manner

**DOI:** 10.7150/ijbs.97425

**Published:** 2024-09-03

**Authors:** Rui Zhang, Pu Chen, Yubo Wang, Zekun Zeng, Huini Yang, Mengdan Li, Xi Liu, Wei Yu, Peng Hou

**Affiliations:** 1 International Joint Research Center for Tumor Precision Medicine of Shaanxi Province and Department of Endocrinology, The First Affiliated Hospital of Xi'an Jiaotong University, Xi'an 710061, P.R. China.; 2 Department of Cardiology, Xi'an Jiaotong University Second Affiliated Hospital, Xi'an 710061, P.R. China.; 3 Department of Pathology, The First Affiliated Hospital of Xi'an Jiaotong University, Xi'an 710061, P.R. China.; 4 BioBank, The First Affiliated Hospital of Xi'an Jiaotong University, Xi'an 710061, P.R. China.

**Keywords:** NSCLC, METTL3, Chemoresistance, ABCC2, m^6^A, YTHDF1

## Abstract

Patients with non-small cell lung cancer (NSCLC) are easily resistant to first-line chemotherapy with paclitaxel (PTX) or carboplatin (CBP). N^6^-methyladenosine (m^6^A) methyltransferase-like 3 (METTL3) has crucial functions in m^6^A modification and tumorigenesis. However, its role in chemoresistance of NSCLC is still elusive. Here, we demonstrated that METTL3 inhibitor STM2457 significantly reduced the IC_50_ values of PTX or CBP in NSCLC cells, and they showed a synergistic effect. Comparing with monotherapy, a combination of STM2457 and PTX or CBP exhibited more potent *in vitro* and *in vivo* anti-tumor efficacy. In addition, we found that ATP binding cassette subfamily C member 2 (ABCC2) was responsively elevated in cytomembrane after PTX or CBP treatment, and targeting METTL3 could reverse this effect. Mechanistically, targeting METTL3 decreased the m^6^A modification of *ABCC2* mRNA and accelerated its mRNA degradation. Further studies revealed that YTHDF1 could bind and stabilize the m^6^A-modified mRNA of *ABCC2*, while YTHDF1 knockdown promoted it mRNA degradation. These results, taken together, demonstrate that targeting METTL3 enhances the sensitivity of NSCLC cells to PTX or CBP by decreasing the cytomembrane-localized ABCC2 in an m^6^A-YTHDF1-dependent manner, and suggest that METTL3 may be a potential therapeutic target for acquired resistance to PTX or CBP in NSCLC.

## Introduction

Lung cancer is the leading cause of cancer mortality among 36 cancer types considered in the worldwide. The approximate number of diagnosed cases was estimated at 236,740 new cases (117,910 in men and 118,830 in women) with 130,180 deaths (68,820 in men and 61,360 in women) in 2022 1-3. The most prevalent kind of lung cancer is non-small cell lung cancer (NSCLC), accounting for approximately 84% of lung cancer cases 4. However, NSCLC has a poor prognosis due to high degree of aggresses and delayed diagnosis 1. The overall survival rate is still poor despite promising treatments including immunotherapy, chemotherapy, radiation-therapy and surgery 5-10.

Chemotherapy is the first-line treatment for NSCLC patients 11. Among them, paclitaxel (PTX) and carboplatin (CBP) are widely used to treat patients with lung cancer 12, 13. PTX promotes the polymerization and stabilization of microtubule and induces cell death by cell cycle arrest in metaphase and chromosome mis-segregation on multipolar spindle 14. CBP can bind DNA bases and change the structure of DNA to form the DNA adducts and cause nuclear DNA and mitochondrion DNA damage, thus inhibiting replication and transcription and eventually inducing cell death 15. However, cells activate the defensive DNA damage response and repair process to mitigate the cytotoxic effects of chemotherapy 16, causing chemotherapy less effective as expected in clinic.

Cancer cells develop resistance to a series of structurally and mechanistically unrelated anti-cancer drugs, which is usually known as multidrug resistance (MDR) 17. In general, there are two major reasons for the development of chemotherapy resistance. Firstly, intrinsic resistance is a consequence of primary genetic mutations especially in the heterogeneous tumor cells. Secondly, acquired resistance is induced by drug treatment and can be due to different mechanisms, including inhibition of cell apoptosis, altered in the drug metabolism and drug targets, enhanced DNA repair, altered uptake pathways and increased expression or activity of drug efflux pumps, eventually resulting in attenuated drug effect or reduced cellular accumulation of drugs 18, 19. One of the most prominent reasons of MDR is high expression of ATP-binding cassette (ABC) transporters. ABC transporters are a class of transporter protein in cytomembrane, which transport their substrates across the cytomembrane, including drugs, nutrients, metabolic products and lipids. Among the known human 48 ABC transporters, at least 20 members are able to export multiple anti-cancer drugs, thereby leading to resistance to chemotherapy 20.

N^6^-Methyladenosine (m^6^A), also known as methylated adenosine at N^6^ position, is a widespread and abundant modification in messenger RNA (mRNA), non-coding RNA (ncRNA) 21 and virus nuclear RNA 18. In mammalian cells, m^6^A modification is reversible and dynamic regulated by m^6^A methyltransferases (METTL3, METTL14, RBM15 and WTAP) and demethylases (FTO and ALKBH5). The m^6^A modified mRNA can be recognized by specific RNA-binding proteins (YTHDF1-3, YTHDC1/2, IGF2BP1-3, etc.), which affect mRNA processing, localization, stability and translation efficiency 22-24. Growing evidence indicates that METTL3 plays a crucial role in tumorigenesis 25, tumor metastasis 26 and resistance to chemotherapy. However, the mechanism by which METTL3 causes the chemotherapy resistance to PTX or CBP is still elusive.

In this study, we demonstrated that targeting METTL3 could improve the response of NSCLC cells to PTX and CBP by a series of *in vitro* and *in vivo* studies. Moreover, we identified ABCC2 as a downstream target of METTL3, and found that ABCC2 was obviously up-regulated in cytomembrane upon chemo-drug exposure. Accordingly, targeting METTL3 down-regulated ABCC2 in cytomembrane, thereby reducing the efflux of chemo-drugs and enhancing their anti-tumor efficacy. Specifically, targeting METTL3 can decrease mRNA stability of *ABCC2* by inhibiting its mRNA m^6^A modification in a YTHDF1-dependent manner. Our data, taken together, suggest that targeting METTL3 will provide a potential therapeutic strategy for chemotherapy-resistant NSCLCs.

## Materials and methods

### Human data sets

The data from The Cancer Genome Atlas (TCGA) database (https://portal.gdc.cancer.gov/) was analyzed to compare gene expression between NSCLC tissues and non-cancerous lung tissues (control subjects).

### Cell culture

NSCLC cell lines A549 and NCI-H460 were purchased from ATCC (American Type Culture Collection, USA). A549 and NCI-H460 cells were cultured in DMEM/Ham's F-12(Gibco) or RPMI-1640 medium supplemented with 10% fetal bovine serum (FBS, Gibco). The cultured cells were kept at 37 ℃ with a humid incubator (Thermo Scientific, USA) in 5% CO_2_.

### Drugs

METTL3 inhibitor STM2457 (HY-134836), carboplatin (HY-17393) and paclitaxel (HY-B0015) were purchased from MedChemExpress (Monmouth Junction, NJ). STM2457 was dissolved by DMSO (Sigma-Aldrich) into a 20 mM stock solution and paclitaxel was dissolved by DMSO into 10 mM stock solution. The carboplatin was dissolved by sterile water into a 25 mM stock solution.

### Short interfering RNAs (siRNAs)

siRNA oligonucleotides targeting METTL3 (si-METTL3 #1 and si-METTL3 #2) or YTHDF1 (si-YTHDF1 #1 and si-YTHDF1 #2) and Control siRNA (si-NC) were purchased from RiboBio (Guangzhou, China), and the sequences of siRNAs used in this study were shown in Supplementary Table S1. Twenty-four hours before transfection, cells were seeded on a 6-well plate with 60% confluence. The next day, cells were transfected with targeted siRNAs at a final concentration of 50 nM using X-treme GENE siRNA Transfection Reagent (Roche Diagnostics) and OPTI-MEM (Invitrogen) according to manufacturer's instructions. Cells were then harvested and analyzed 48-72 h post transfection.

### Cell viability assay

Cells (1500/well) were cultured in 96-well plates and treated with STM2457 and PTX or CBP, individually or in combination at the indicated concentrations and time points. Cell viability and the half maximal inhibitory concentration (IC_50_) value was then calculated as previously mentioned 27.

### Colony formation assay

Cells (2000/well) were seeded into 12-well plates for culturing and then treated with STM2457 and PTX or CBP, individually or in combination at the indicated concentrations for 10 to 14 days. Cells were fixed using methyl alcohol for 15 min, colony was washed and stained with crystal violet for 15 min, and counted under an inverted microscope. Each assay was carried out in triplicate.

### Cell apoptosis assay

Cells were collected and washed with ice-cold PBS for twice. At least 10,000 cells were collected from each sample. The 100 μL binding buffer, Annexin Ⅴ-FITC and PI were added to cells according to the manufacturer's instructions of apoptosis kit (4A BIOTECH, #FXP018). Cell apoptosis was then detected by flow cytometry (BD Biosciences, NJ).

### Cell cycle assay

Cells were seeded to 6-well plate and cultured in serum-free medium for 12 h after cells attachment to plates. Cells were then treated with STM2457 and PTX or CBP, individually or in combination at the indicated concentrations and time points. Next, cells were fixed with 75% cold methanol for at least 2 h. After staining the cells with PI, flow cytometry was used to analyze the distribution of cell cycle.

### RNA isolation and quantitative RT-PCR (qRT-PCR)

The procedures about RNA extraction, cDNA synthesis and qRT-PCR assays were performed as described previously 28. The mRNA expression was normalized to *β-actin* or *18S* rRNA. Each sample was repeated in triplicate. The primer sequences were listed in Supplementary Table S2.

### Western blotting analysis

The protocol was similarly performed as described previously 29. Briefly, cells were cultured and treated with the specific conditions. Cells were washed with cold PBS twice and lysed with RIPA lysis buffer (NCM, #WB 3100) supplemented with phosphatase inhibitors and PMSF (Zhhcbio, #PL012-1) for 25 min. Following centrifugation at 12,000 rpm at 4°C, the supernatant of cell lysate was collected and protein concentration was then measured by Nanodrop. Protein extractions were supplied on 10% SDS-PAGE electrophoresis, and then transferred into PVDF membranes (Sigma-Aldrich, #63116500). After 2 h blocking in Bovine Serum Albumin (Amresco, #9048-46-8), the membranes were incubated with primary antibodies at 4℃ overnight, followed by being incubated with corresponding secondary antibodies. Immunoblotting signals were captured and analyzed by Tanon 5200 Multi fully automatic chemiluminescence system. The information of antibodies was listed in Supplementary Table S3.

### Membrane and cytosol protein extraction

The membrane and cytosol proteins were extracted by the Membrane and Cytosol Protein Extraction Kit (Beyotime, #P0033) based on the manufacturer's instructions. Briefly, cells were washed with cold PBS twice and scraped off by scrapers, followed by centrifugation at 600 g at 4°C for 5 min. Cells were then lysed with Buffer A supplemented with PMSF for 15 min on the ice. Following freezing in the liquid nitrogen and melting at room temperature for twice, cells were centrifuged at 700 g at 4°C for 10 min and the supernatant was harvested which contains the cytosol protein. After centrifuging at 14000 g at 4°C for 30 min and removing all the supernatant, the sediment was lysed with Buffer B supplemented with PMSF and vortexed for 5 sec, then lysis was stand in the ice for 5-10 min. Finally, the lysis was centrifuged at 14000 g at 4°C for 5 min, and the supernatant containing membrane proteins were harvest. The cytosol and membrane proteins were subjected to western blotting analysis to measure the expression levels of ABCC2.

### Immunofluorescence (IF) staining

Cells were seeded on sterile glass slides at 24-well for 24 h. Cell membrane was then stained using Cell Plasma Membrane Staining Kit with Dil (Red Fluorescence) (Beyotime, #C1991S) according to manufacturer's instructions. Next, cells were fixed with cold methanol for 10 min and permeated with 0.3% Triton-X (Beyotime, #P0096). The slides were incubated with primary antibodies at 4℃ overnight and fluorescent secondary antibodies (1:2000; Invitrogen) for 2 h at room temperature in the dark. DAPI was used to stain the nucleus. The stained slides were stored in glycerol and imaged using a laser scanning confocal microscope (Leica, Wetzlar, German).

### Evaluation of ABCC2 mRNA stability

Cells were seeded onto 12-well plates and treated with the indicated conditions. To measure mRNA stability of *ABCC2* in NSCLC cells, 10 μg/mL actinomycin D (MCE, #HY-17559) was added to culture solution and incubated for different time points. Next, cells were harvested and RNA was then isolated. *ABCC2* mRNA expression was measured by qRT-PCR in specific time points and normalized to *18S* rRNA.

### Dual-luciferase reporter system

The plasmids used in luciferase experiments were as follows: (1) pRL-TK vector, for the expression of Renilla luciferase, and (2) a plasmid encoding an ATG start codon followed by 60 bp surrounding the *ABCC2* m^6^A site (5'-CTG AAG GAA GAC GAA GAA CTA GTG AAA GGA CAA AAA CTA ATT AAG AAG GAA TTC ATA GAA-3') and a linker followed by firefly luciferase based on PCI-neo vector, and (3) a point-mutant plasmid in which “GGACAA” was mutant to “GGTCAA”. These plasmids were pooled together and transfected to cells according to instructions. Cells were treated with the indicated drugs or siRNAs in 24-well plates for 24 h, and transfected with dual-luciferase reporter plasmids. The luciferase intensity was then measured by Dual-Glo luciferase Assay System (Promega) based on the manufacturer's instructions. Luciferase activity was defined as the ratio of firefly luciferase activity versus Renilla luciferase activity.

### Methylated RNA immunoprecipitation and qPCR (MeRIP-qPCR)

The protocol of MeRIP was described previously 30. In brief, poly(A) RNA was purified from 40 μg RNA and fragmented into ~150 nt using magnesium RNA fragmented buffer (NEB, #6150S), then fragmented RNA was concentrated by glycogen and ethanol precipitation. A tenth of the RNA was sub-packaged as the input control and the rest RNA was incubated with 5 μg anti-m^6^A antibody in IP buffer supplemented with RNase inhibitor (Promega, #N2611) and RVC (Sigma-Aldrich, #R3380) for 2 h at 4℃. Protein A/G UltraLink Resins (Pierce, #53132) were prewashed twice by 1 × IP buffer containing BSA (Sigma-Aldrich, 0.5 mg/mL,** #**9048-46-8), and then mixed with the RNA-antibody complex at 4℃ for 2 h. Next, the methylated mRNAs were eluted by N^6^-methyladensine 5'-monophosphate disodium salt (USA, #HY-111926) precipitated with ^1^/_10_ of 3 M sodium acetate and 5 μg of glycogen in 2.5 volumes of 100% ethanol at -80℃ overnight. Further enrichment was calculated by qPCR, and the corresponding m^6^A enrichment in each sample was calculated by normalizing to the input. The primers used for MeRIP-qPCR were presented in Supplementary Table S4.

### Flow cytometric detection of ABCC2 protein expression

Flow cytometry was performed to detect protein levels of ABCC2 as described previously 31. Briefly, A549 and NCI-H460 cells were cultured for 48 h in specialized condition. Cells were collected and washed with ice-cold PBS, and then fixed with 4% paraformaldehyde for 10-15 min. Next, cells were permeated by tween-20 for 10 min following incubating cells in the instructional primary antibodies for 30 min at 4℃. After washing with PBS, cells were incubated in fluorescent secondary antibody (Goat Anti-Rabbit IgG H&L (AF488)) for 30 min at room temperature. Finally, cells were washed three times with PBS, and then responded in 200 μL PBS to detected the mean fluorescence intensity of ABCC2, or resuspended in 500 μL 1% paraformaldehyde overnight.

### RNA immunoprecipitation (RIP)

Cells were collected at 80-90% of confluence, and washed by PBS and resuspended with lysis buffer containing RNA enzyme inhibitors (1: 200) and protease inhibitors (1: 100). The mix were then incubated on ice for 5 min. Cell lysate was divided into three groups (input, IP and IgG). IgG antibody (5 μg) and anti-YTHDF1 antibody (5 μg) were incubated with cell lysates of IgG and IP groups at 4 °C for 16 h, respectively. Meanwhile, 50-100 μL protein A/G magnetic beads (MCE, #HY-K0202) were washed twice and resuspended with lysis buffer. Next, cell lysates containing RNA-protein complexes were incubated with the protein A/G magnetic beads at 4 °C for 3 h. After proteinase K digestion, protein-bound RNAs were separated by phenol/chloroform/isoamyl alcohol (25:24:1), and extracted by glycogen/sodium acetate/100% (1:10:500) at -80°C for 3 h. The RNAs were then washed by 80% precooled ethanol and dissolved by RNase-free water. The protein-bound RNAs were detected by qRT-PCR.

### Animal studies

Four to five-week-old male nude mice were obtained from Huachuang Sino Pharmatech Co., Ltd. (Taizhou, Jiangsu, China), and feed with sterilized food and water and bred in a specific pathogen-free (SPF) environment. To establish xenograft tumor model, NCI-H460 cells were collected and resuspended at a density of 5 × 10^6^ cells per 100 μL PBS and injected subcutaneously into nude mice. When tumor volume reached 80-90 mm^3^, mice were divided into two batches, each batch was then randomly divided into four groups (five mice/group) and the treatment was begun. In the first batch of mice, 30 mg/kg STM2457 and 3 ng/kg PTX were administered individually or in combination via intraperitoneal injection, and equal volume of vehicle was administered as the control. In the second batch of mice, 30 mg/kg STM2457 and 30 mg/kg CBP were administered individually or in combination via intraperitoneal injection, with equal volume of vehicle as the control. STM2457 was administered every day, while PTX and CBP were administered once every two days for continuous two weeks. During the treatment, tumor and body weight of mice were measured every four days, and tumor volume was calculated as length × width ^2^ × 0.5. At the end of experiments, mice were sacrificed and tumors were isolated for the following studies. One-way ANOVA was used to statistically analyze tumor size and tumor weight. The study was approved by the Animal Ethics Committee of Xi' an Jiaotong University.

### Immunohistochemistry (IHC) and H&E staining

The protocols for IHC staining of xenograft tumors and H&E staining of kidney and liver tissues were described previously 29.

### Drug safety evaluation

The blood samples of mice were stand for 10 min, and then centrifuged at 1000 rpm for 10 min at room temperature to isolate serum. Next, the serum samples were used to measure the levels of blood urea nitrogen (BUN), aspartate aminotransferase (AST), alanine transaminase (ALT) and serum creatinine (CRE) by the corresponding kits (#C013-2-1, #C010-2-1, #C009-2-1 and #C011-2-1, Naning Jiancheng Bioengineering Institute) as described previously 27.

### Statistical analysis

Gene expression in cancer and normal tissues was compared by an unpaired *t* test, while gene expression in paired samples was compared by a paired *t* test. Nonlinear regression (curve fit) was used to analyzed the IC_50_ values of drugs. Two-way ANOVA was used to compare the mRNA stability data. All statistical analyses were calculated by SPSS. *P* <0.05 was considered statically significant.

## Results

### METTL3 inhibition sensitizes NSCLC cells to PTX and CBP

We first evaluated *METTL*3 expression in lung cancer tissues and non-cancerous lung tissues (control subjects) using The Cancer Genome Atlas (TCGA) database. The results showed that METTL3 was substantially up-regulated in both lung adenocarcinoma (LUAD) and lung squamous cell carcinoma (LUSC) patients compared with control subjects (Supplementary Figure S1). To determine whether *METTL3* expression correlates with the chemosensitivity, we treated A549 and NCI-H460 cells with a series dose of METTL3 inhibitor STM2457, and measured their half maximal inhibitory concentration (IC_50_). The results showed that the IC_50_ values of STM2457 in A549 and NCI-H460 cells were 14.06 μM and 48.77 μM, respectively (Supplementary Figure S2), indicating the anti-tumor effect of STM2457 in NSCLC cells.

**Figure 1:**
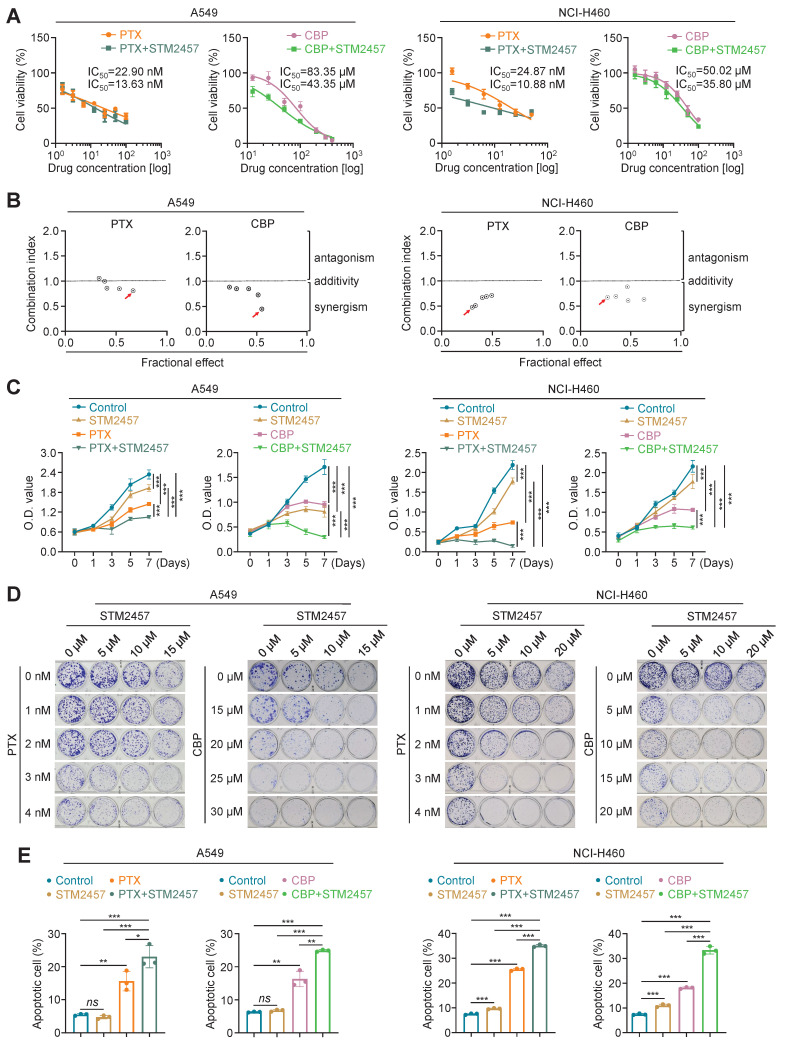
** The sensitizing effect of METTL3 inhibitor STM2457 on PTX and CBP *in vitro*. (A)** NSCLC cells were treated with the indicated concentrations of PTX or CBP individually or in combined with 5 μM STM2457 in A549 cells and 20 μM STM2457 in NCI-H460 cells for 48 h. MTT assay was then performed to evaluated cell viability, and Reed-Muench method was used to calculated the IC_50_ values. **(B)** The dose-dependent effect between STM2457 and PTX or CBP in A549 and NCI-H460 cells was assessed and the combination index (CI) values were then calculated using the Chou-Talalay dose-effect method. CI<1, CI=1 and CI>1 represented the synergism, additivity and antagonism of two drugs, respectively. The combinations of 5 μM STM2457 with 3 nM PTX or 20 μM CBP in A549 cells and the combinations of 20 μM STM2457 with 5 nM PTX or 30 μM CBP in NCI-H460 cells were shown as the “red arrow”. **(C)** A549 cells were treated with 5 μM STM2457 individually or in combination with 3 nM PTX or 20 μM CBP for 7 days. NCI-H460 cells were treated with 20 μM STM2457 individually or in combination with 5 nM PTX or 30 μM CBP for 7 days. Cell viability was then calculated by MTT assay. **(D)** A549 and NCI-H460 cells were treated with the indicated dose of STM2457 and PTX or CBP, individually or in combination, and their effect on cell colony formation ability was then evaluated. **(E)** A549 and NCI-H460 cells were treated with 5 μM or 20 μM STM2457 individually or in combination with 5 nM PTX or 30 μM CBP. After a 48-h treatment, the apoptotic cells were quantified by flow cytometry. The data were presented as the mean ± SD. *, *P* <0.05; **, *P* <0.01; ***, *P* <0.001;* ns*, no significance.

To determine the effect of STM2457 on the response of NSCLC cells to PTX or CBP, we treated A549 and NCI-H460 cells with PTX or CBP, individually or in combination with STM2457 (5 μM for A549 cells and 20 μM for NCI-H460 cells) for 48 h. The results showed that STM2457 obviously decreased the IC_50_ values of PTX or CBP in A549 and NCI-H460 cells (Fig. 1A). In addition, we treated A549 and NCI-H460 cells with different concentrations of STM2457 and PTX or CBP, and calculated their combination index (CI) using Chou-Talalay method. As shown in Fig. 1B, the combination of STM2457 and PTX or CBP showed a considerable synergistic effect at the indicated concentrations. As supported, the combination of STM2457 and PTX or CBP significantly inhibited the proliferation of A549 and NCI-H460 cells compared with PTX or CBP treatment alone (Fig. 1C). Also, we found the synergetic inhibitory effect of STM2457 and PTX or CBP on colony formation ability of A549 and NCI-H460 cells in a dose-dependent manner (Fig. 1D and Supplementary Figure S3). Next, we similarly treated A549 and NCI-H460 cells with PTX or CBP, individually or in combination with STM2457, and used flow cytometry to evaluate their effect on cell apoptosis. As expected, PTX or CBP significantly induced cell apoptosis, while this effect was more obvious when combined with STM2457 (Fig. 1E and Supplementary Figure S4A). Also, we detected the effect of PTX or CBP treatment alone or in combination with STM2457 on the levels of apoptosis-related markers such as BCL-2, BAX and cleaved-caspase 3 in A549 and NCI-H460 cells. The results further supported the above conclusions showing that the combination of PTX or CBP with STM2457 had more significant effect on their levels compared with monotherapy (Supplementary Figure S4B).

Considering that PTX has been demonstrated to induce cell cycle arrest in tumor cells 32, 33, thus we evaluated the effect of PTX and STM2457, individually or in combination, on cell cycle distribution in A549 and NCI-H460 cells. The results showed that PTX induced G2/M cell cycle arrest compared with the control, while this effect was further enhanced when combined with STM2457 (Supplementary Figure S5A). Meanwhile, we also found that the levels of cyclin B1, a pivotal marker of G2/M cell cycle, were up-regulated in A549 and NCI-H460 cells upon PTX treatment, and its production was further elevated when combined with STM2457 (Supplementary Figure S5B). In contrast to PTX, CBP can bind to DNA and form DNA adducts, causing cytotoxicity through DNA damage 34. Thus, we treated A549 and NCI-H460 cells with CBP and STM2457, individually or in combination, and evaluated their effect on DNA damage by detecting γH2AX foci using immunofluorescence staining. The results showed that the combination of STM2457 and CBP obviously increased the number of γH2AX foci compared with monotherapy (Supplementary Figure S6A). This was also supported by the results of western blotting analysis showing that the combination of STM2457 and CBP further elevated the expression of γH2AX compared with each treatment alone (Supplementary Figure S6B). Our data, taken together, indicate that targeting METTL3 sensitizes NSCLC cells to PTX or CBP.

### METTL3 inhibition improves the* in vivo* anti-tumor efficacy of PTX and CBP

**Figure 2:**
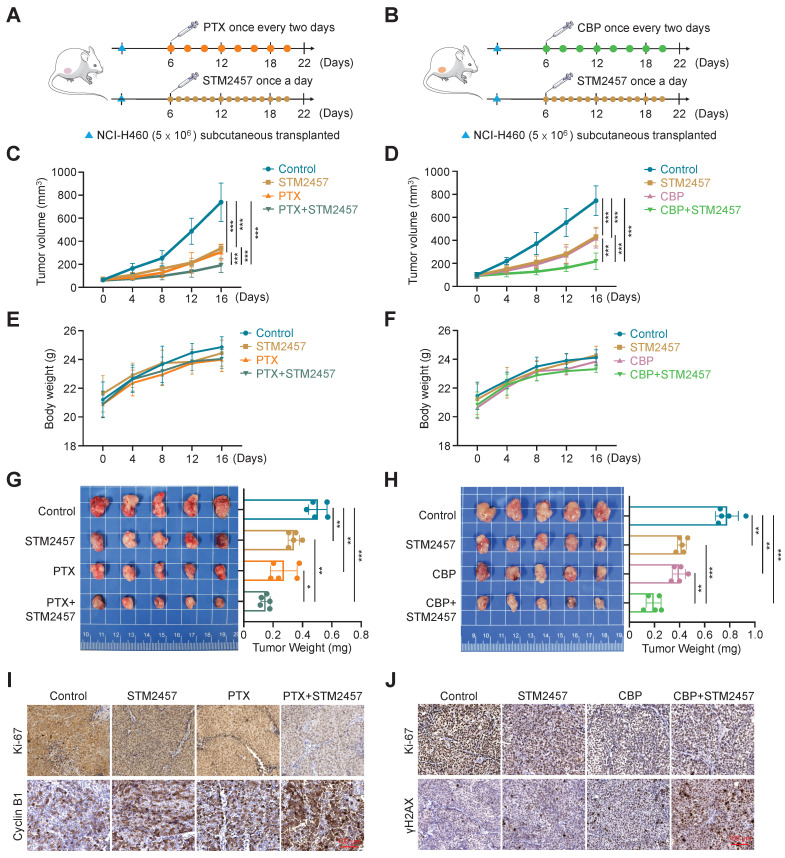
** The sensitizing effect of METTL3 inhibitor STM2457 on PTX and CBP *in vivo*. (A-B)** The experiment procedure of NCI-H460 cell-derived xenograft tumor model. Briefly, tumor-bearing nude mice were grouped randomly (n =5/group) and treated with STM2457 (30 mg/kg, once a day) and PTX (3 ng/kg, once every two days) or CBP (30 mg/kg, once every two days), individually or in combination, for 2 weeks. **(C-D)** The growth curves of xenograft tumors with the indicated treatments. Day 0 represented the day of drugs injection. **(E-F)** The growth curves of body weight in the indicated groups.** (G-H)** The left panel showed the images of dissected tumors from the indicated groups, and tumor weight was shown in the right panel.** (I-J)** The representative sections from the indicated tumors were subjected to IHC staining using the corresponding antibodies. Scale bar, 100 μm. The data were presented as the mean ± SD. *, *P* <0.05; **, *P* <0.01; ***, *P* <0.001.

To evaluate the effect of targeting METTL3 on the *in vivo* efficacy of PTX and CBP, we established the xenograft tumor model by subcutaneously injecting 5 × 10^6^ NCI-H460 cells and randomly divided mice into two batches (4 groups/batch) when the tumor volume reached 80-90 mm^3^. One batch of mice were further randomly divided into four groups and intraperitoneally administrated with STM2457 (30 mg/kg) each alone or in combination with PTX (3 ng/kg) (Fig. 2A), while another batch of mice were also divided into four groups and treated with STM2457 (30 mg/kg) and CBP (30 mg/kg) each alone or in combination (Fig. 2B). STM2457 was administered once per day, while PTX and CBP were administered every two days for a continuous two-week period. The results showed that STM2547-, PTX- or CBP-treated tumors grew slowly compared to control tumors, while a combination of STM2547 and PTX or CBP exhibited more obviously growth-inhibitory effect compared to each treatment alone (Fig. 2C-D). During the treatment, we did not find significant difference in body weight among these groups (Fig. 2E-F). After 16 days of exposure to drugs, we sacrificed tumor-bearing mice to harvest the xenograft tumors and measured the tumors weight. As expected, we found that a combination of STM2457 and PTX or CBP further decreased tumor weight compared with PTX or CBP treatment alone (Fig. 2G-H). This was also supported by the results of Ki-67 staining in the above xenograft tumor tissues showing that a combination of STM2457 and PTX or CBP caused a more significant decrease in the levels of Ki-67 compared with each treatment alone (Fig. 2I-J). Besides, we found that, compared with monotherapy, the levels of cyclin B1 or γH2AX were obviously up-regulated in the tumors treated with a combined therapy of STM2457 and PTX or CBP (Fig. 2I-J), further supporting the above conclusions. Importantly, we failed to find any changes in serological indicators of liver and kidney function, including ALT, AST, BUN and CRE, in mice with different treatments (Supplementary Figure S7A-B), suggesting that the above treatment strategies did not cause severe hepatorenal toxicity. This conclusion was also supported by H&E staining (Supplementary Figure S7C-D). Collectively, our data further demonstrate that the combination of STM2457 with PTX or CBP may be a safe and potential therapeutic strategy for chemotherapy resistant NSCLC.

### PTX or CBP up-regulates cytomembrane-localized ABCC2 in NSCLC cells

**Figure 3:**
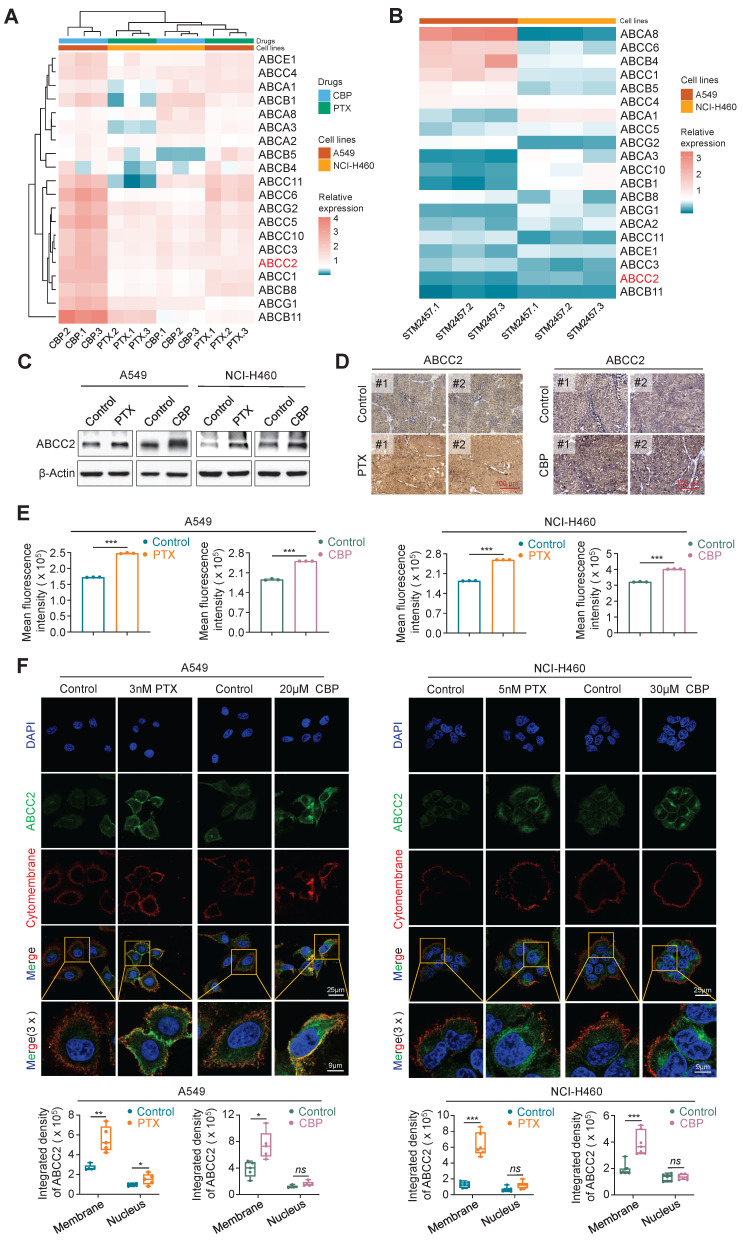
** PTX or CBP-induced up-regulation of ABCC2 in the cytomembrane. (A)** A549 cells were treated 3 nM PTX or 20 μM CBP and NCI-H460 cells were treated 5 nM PTX or 30 μM CBP for 48 h. The mRNA expression of 20 members of ABC transporters was then measured by qRT-PCR assay. **(B)** A549 and NCI-H460 cells were treated with 5 μM and 20 μM STM2457, respectively. qRT-PCR was then performed to determine their effect on the mRNA expression of the above ABC transporters. *β-actin* was used as the internal control for normalization. **(C)** A549 cells were treated with 3 nM PTX or 20 μM CBP and NCI-H460 cells were treated with 5 nM PTX or 30 μM CBP for 48 h. The protein expression of ABCC2 was measured by western blotting analysis. β-Actin was used as a loading control.** (D)** The representative sections from the indicated tumors were subjected to IHC staining using anti-ABCC2 antibody. Scale bar, 100 μm. **(E)** A549 cells were treated with 3 nM PTX or 20 μM CBP and NCI-H460 cells were treated with 5 nM PTX or 30 μM CBP for 48 h. The protein expression of ABCC2 was then measured by flow cytometry. **(F)** The representative immunofluorescence images of ABCC2 in A549 and NCI-H460 cells treated with the indicated dose of PTX or CBP for 48 h (upper panels). Blue color represents the staining of nuclei, green color represents the staining of ABCC2 and red color presents the staining of cytomembrane. Scale bars, 25 μm. The integrated density of ABCC2 in the cytomembrane and nucleus was then analyzed and showed in the lower panels. The data were presented as the mean ± SD. *, *P* <0.05; **, *P* <0.01; ***, *P* <0.001; *ns*, no significance.

Numerous studies have shown that the aberrant expression of ATP-binding cassette (ABC) transporters directly affects the cytotoxic effects of chemotherapeutic agents 35, 36. In turn, chemotherapeutic agents can also up-regulate the expression of some of ABC transporters 37-39. To explore whether ABC transporters involved in the process of chemoresistance in NSCLC cells, we first treated A549 and NCI-H460 cells with PTX or CBP, and determined their effect on the expression of 20 ABC transporters, which have been demonstrated to participate in chemotherapy resistance 20. The results found that ABCC2, ABCC3, ABCC4, ABCC5, ABCC10 and ABCG2 could be significantly up-regulated by both PTX and CBP (Fig. 3A). To elucidate the molecular mechanism by which targeting METTL3 sensitizes PTX and CBP, we treated A549 and NCI-H460 cells with STM2457 to determine its effect on the expression of these 20 members of ABC transporters. The results showed that STM2457 significantly decreased the expression of ABCB11, ABCC2, ABCC3, ABCE1 and ABCC11 (Fig. 3B). Next, among the above molecules that were both up-regulated by PTX or CBP and down-regulated by STM2457, we mainly focused on ABCC2, also known as multi-drug resistance protein 2 (MRP-2), because there is evidence showing that it may cause the resistance of cancer cells to PTX and CBP 40, 41.

We next analyzed mRNA expression of ABCC2 in lung cancers and noncancerous lung tissues (control subjects) using The Cancer Genome Atlas (TCGA) RNA-Seq dataset, and found that its expression was significant up-regulated in LUADs and LUSCs compared with control subjects (Supplementary Figure S8). As supported, we treated A549 and NCI-H460 cells with PTX or CBP for 48 h, and evaluated their effect on the expression of ABCC2 by qRT-PCR and western blotting assays. The results showed that both PTX and CBP significantly increased the mRNA and protein levels of ABCC2 (Fig. 3C and Supplementary Figure S9). In addition, we also found elevated expression of ABCC2 in PTX- or CBP-xenograft tumors compared with control tumors by immunohistochemical staining (Fig. 3D). This was also supported by the results of flow cytometry assay (Fig. 3E). Notably, we observed that either PTX or CBP dramatically increased the levels of ABCC2 in the cytomembrane compared with the control by immunofluorescence assay (Fig. 3F). These findings, taken together, suggest that drug-responsive up-regulation of ABCC2 may contribute to the resistance of NSCLC cells to PTX and CBP.

### Targeting METTL3 down-regulates the expression of ABCC2 in cytomembrane

**Figure 4:**
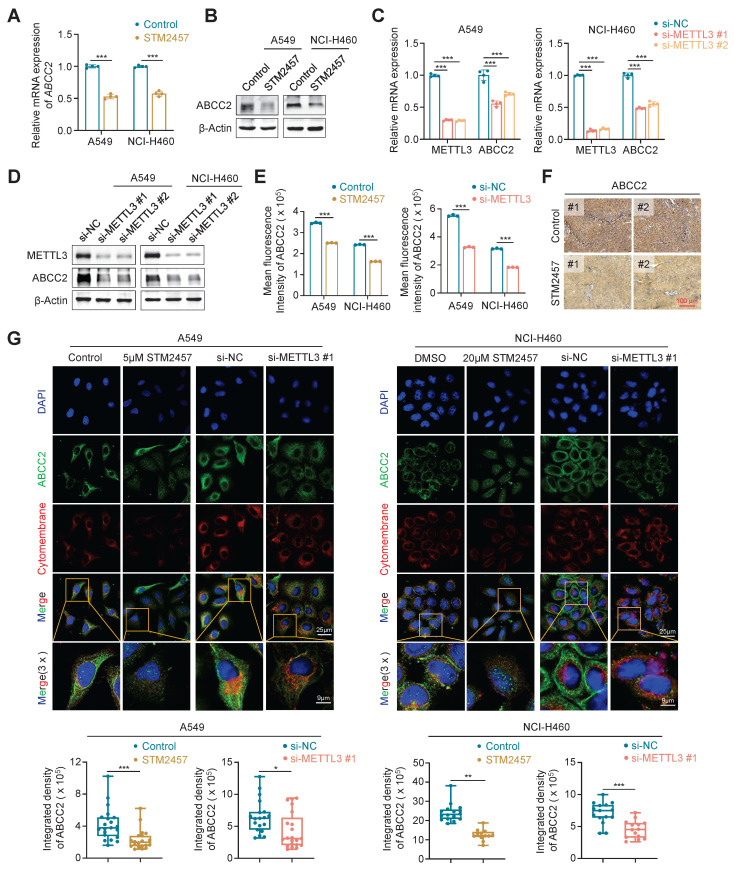
** Down-regulation of cytomembrane-localized ABCC2 by METTL3 inhibition.** A549 and NCI-H460 cells were treated with 5 μM and 20 μM STM2457, respectively. qRT-PCR **(A)** and western blotting assays **(B)** were then performed to determine their effect on mRNA and protein expression of ABCC2. METTL3 was knocked down in A549 and NCI-H460 cells by siRNAs targeting METTL3 (si-METTL3 #1 and si-METTL3 #2). qRT-PCR **(C)** and western blotting **(D)** assays were then performed to detect mRNA and protein expression of ABCC2. **(E)** A549 and NCI-H460 cells were treated with 5 μM and 20 μM STM2457 for 48 h, respectively (left panel), or METTL3 was knocked down in these two cell lines (right panel). ABCC2 expression was then quantified by flow cytometry. **(F)** The representative sections from the indicated tumors were subjected to IHC staining using anti-ABCC2 antibody. Scale bar, 100 μm. **(G)** The representative immunofluorescence images of ABCC2 in STM2457-treated or METTL3-knockdown A549 and NCI-H460 cells (upper panels). Blue color represents the staining of nuclei, green color represents the staining of ABCC2 and red color represents the staining of cytomembrane. The integrated density of ABCC2 in cytomembrane was then analyzed and showed in the lower panel. *β-actin* was used as the internal control for qRT-PCR, and β-Actin was used as a loading control for western blotting analysis. The data were presented as the mean ± SD. *, *P* <0.05; **, *P* <0.01; ***, *P* <0.001.

To determine whether METTL3 inhibition can sensitize NSCLC cells to PTX or CBP by regulating ABCC2 expression in cytomembrane, we treated A549 and NCI-H460 cells with STM2457 or knocked down METTL3 in these cells, and evaluated their effect on the expression of ABCC2 by qRT-PCR, western blotting, flow cytometry and immunofluorescence assays. The results showed that STM2457 significantly down-regulated mRNA and protein levels of ABCC2 in A549 and NCI-H460 cells compared to the control (Fig. 4A-B), which was consistent with our above results. We further separated the cytomembrane and cytosol proteins after treating NSCLC cells with STM2457, and performed western blotting analysis to detect the expression levels of ABCC2. The results showed that the expression of ABCC2 in cytomembrane was significantly decreased upon STM2457 treatment in A549 and NCI-H460 cells, while ABCC2 was barely expressed in cytosol (Supplementary Figure S10). Similarly, knockdown of METTL3 also decreased the expression of ABCC2 at both mRNA and protein levels (Fig. 4C-D), which was further validated by the results of flow cytometry assay (Fig. 4E) and immunohistochemical staining of ABCC2 in STM2457-treated tumors and control tumors (Fig. 4F). In addition, immunofluorescence assay showed that STM2457 or METTL3 knockdown decreased the levels of cytomembrane-localized ABCC2 by 1.5 to 2-fold in A549 and NCI-H460 cells compared with the control (Fig. 4G). To further confirm the expression level of ABCC2 was directly related to the sensitivity of paclitaxel or carboplatin, we knocked down ABCC2 in A549 and NCI-H460 cells (Supplementary Figure S11A), and further treated them with the indicated doses of PTX or CBP. Next, we performed the MTT assays to evaluated cell viability. The results showed that ABCC2 knockdown reduced the IC_50_ values of PTX and CBP in A549 and NCI-H460 cells (Supplementary Figure S11B-C), indicating that reduced ABCC2 expression improved cellular response to paclitaxel or carboplatin. Taken together, our results suggest that targeting METTL3 to reduce ABCC2 expression in cytomembrane may be one of major causes for improving the chemosensitivity of NSCLC cells to PTX or CBP.

### METTL3 inhibition decreases ABCC2 mRNA stability in an m^6^A-dependent manner

**Figure 5:**
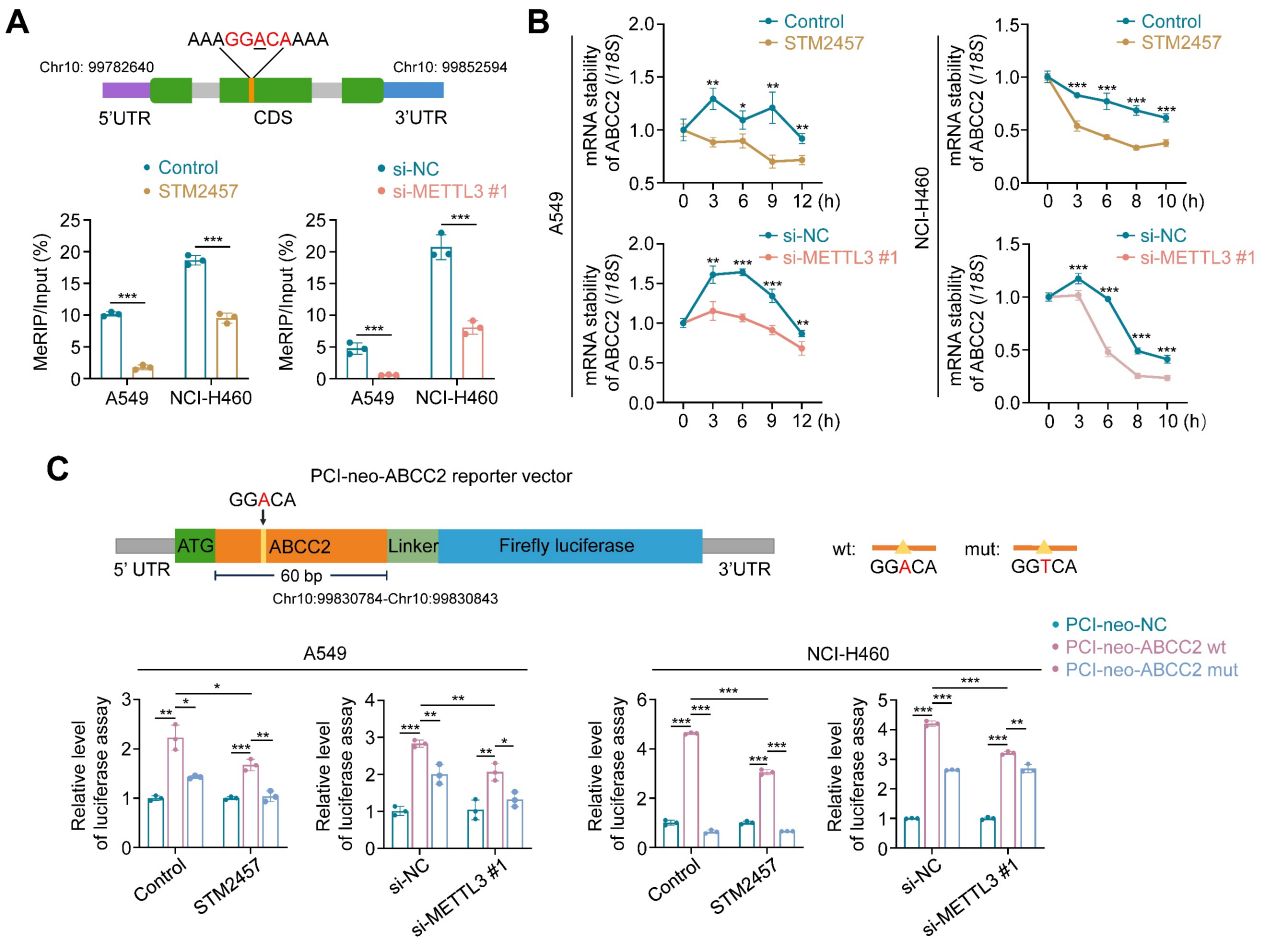
** Induction of *ABCC2* mRNA degradation by METTL3 inhibition in a m^6^A-dependent manner. (A)** Schematic m^6^A-containing sequence in the CDS of *ABCC2* mRNA (upper panel). The site of m^6^A was underlined. A549 and NCI-H460 cells were treated with 5 μM and 20 μM STM2457, respectively, or METTL3 was knocked down in these two cell lines. After 48 h, the m^6^A levels of *ABCC2* were detected by MeRIP-qPCR (lower panel) (n =3, paired *t* test). **(B)** A549 and NCI-H460 cells were pre-treated with the same conditions as above for 48 h, and further treated with 10 μg/mL actinomycin D in the indicated time points. qRT-PCR was then performed to determine their effect on mRNA expression of *ABCC2*, with *18S* rRNA as the normalization control. **(C)** Schematic dual-luciferase reporter plasmids containing wild-type or mutant m^6^A site were showed in the upper panel. A549 and NCI-H460 cells were treated with 5 μM and 20 μM STM2457, respectively, or METTL3 was knocked down in these two cell lines. After 24 h, the above cells were transfected with PCI-neo-ABCC2-wt plasmid containing wild type m^6^A site or PCI-neo-ABCC2-mut plasmid containing mutant (A→T) m^6^A site or PCI-neo-NC plasmid for 36 h. Dual-luciferase reporter assay was then performed to measure the luciferase intensity (lower panel). Data were presented as mean ± SD. *, *P* < 0.05; **, *P* < 0.01; ***, *P* < 0.001.

We next attempted to reveal the molecular mechanisms by which METTL3 regulated the expression of ABCC2 in NSCLC cells. Considering that METTL3 is a m^6^A methyltransferase and functions as a m^6^A writer to modulate mRNA biogenesis, decay and translation via m^6^A modification 21, 23, we thus speculated that ABCC2 might be a potential target of METTL3. Using several online databases, including SRAMP (http://www.cuilab.cn/sramp), RMVar (https://rmvar.renlab.org/) and RMBase (https://rna.sysu.edu.cn/rmbase/index.php), we found a putative m^6^A methylation site (5'-GGACA-3') located in the coding sequences (CDS) of *ABCC2* mRNA, and performed methylated RNA immunoprecipitation (MeRIP)-qPCR assay to confirm that STM2457 or METTL3 knockdown remarkably decreased the levels of m^6^A methylation at this putative site by 5.7 or 8.1 times (Fig. 5A).

To determine the effect of METTL3 inhibition on mRNA stability of ABCC2, we treated STM2457-pretreated or METTL3-knockdown A549 and NCI-H460 cells with 10 μg/mL actinomycin D to inhibit mRNA transcription. The results showed that the decay rate of *ABCC2* mRNA was significantly accelerated upon STM2457 or METTL3 knockdown (Fig. 5B). We then constructed the luciferase reporter plasmids containing wild-type m^6^A motif (GGACA) or mutant m^6^A motif (GGTCA) (Fig. 5C, upper panel), and demonstrated that ectopic expression of ABCC2 wild-type reporter plasmid significantly increased the intensity of firefly luciferase in both STM2457-treated or METTL3-knockdown A549 and NCI-H460 cells and control cell, while ectopic expression of ABCC2 mutant reporter plasmid substantially alleviated this effect (Fig. 5C). In addition, we expectedly found that STM2457 or METTL3 knockdown dramatically decreased the fluorescence intensity of ABCC2 wild-type reporter plasmid, but not mutant one, compared with the controls (Fig. 5C). Collectively, our data indicates that METTL3 inhibition leads to the mRNA instability of *ABCC2* by reducing m^6^A levels in its mRNA.

### YTHDF1 promotes ABCC2 mRNA stability by recognizing and binding to the m^6^A site in the its coding sequence (CDS) region

**Figure 6:**
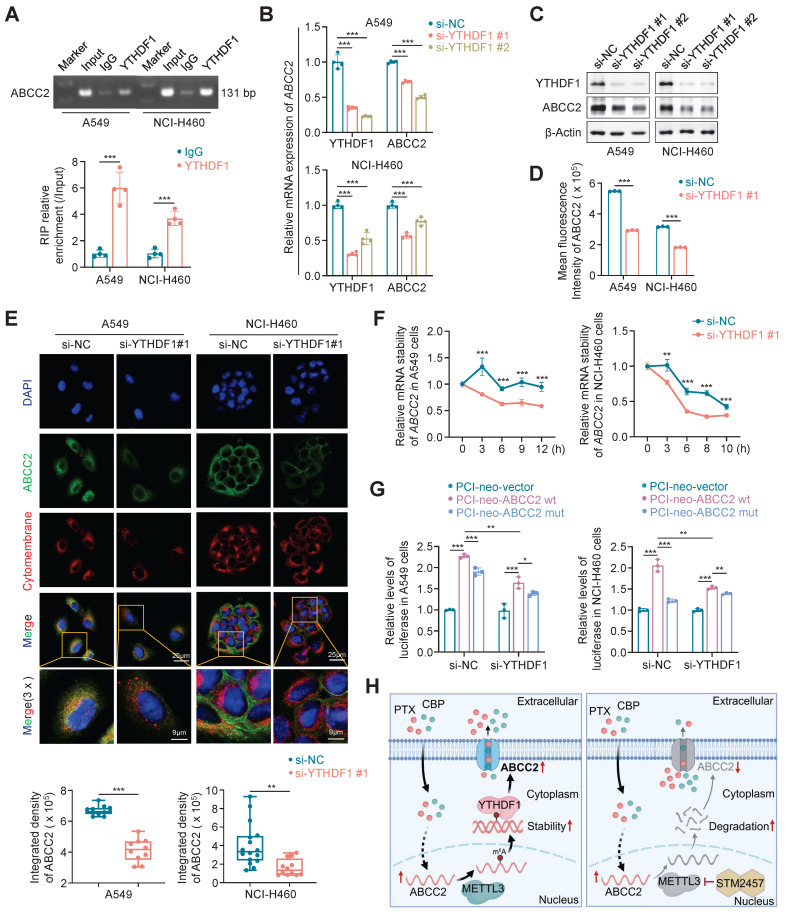
** YTHDF1 stabilizes *ABCC2* mRNA. (A)** Direct binding of YTHDF1 to *ABCC2* mRNA was validated by RIP assays and agarose electrophoresis in A549 and NCI-H460 cells. YTHDF1 was knocked down in A549 and NCI-H460 cells, and qRT-PCR **(B)** and western blotting **(C)** assays were then performed to determine its effect on the mRNA and protein expression of ABCC2. *β-actin* was used as the internal control for qRT-PCR, and β-Actin was used as a loading control for western blotting analysis. **(D)** ABCC2 expression in YTHDF1-knockdown A549 and NCI-H460 cells and control cells was quantified by flow cytometry. **(E)** The representative immunofluorescence images of ABCC2 in YTHDF1-knockdown A549 and NCI-H460 cells and control cells (upper panels). Blue color represents the staining of nuclei, green color represents the staining of ABCC2 and red color represents the staining of cytomembrane. The quantification was shown in the lower panel. **(F)** YTHDF1 were knocked down in A549 and NCI-H460 cells. After 48 h, cells were treated with 10 μg/mL actinomycin D in the indicated time points. The mRNA levels of *ABCC2* were monitored over time using qRT-PCR to assess the decay rate. *18S* rRNA was used as the normalization control. **(G)** YTHDF1 was knocked down in A549 and NCI-H460 cells. After 24 h, cells were transfected with PCI-neo-ABCC2 plasmid containing wild type m^6^A site or PCI-neo-ABCC2 mut plasmid containing mutant (A→T) m^6^A site or PCI-neo-NC plasmid as control. Dual-luciferase reporter assay was then performed to measure the luciferase intensity. **(H)** A schematic model by which METTL3 inhibitor STM2457 enhances the chemosensitivity of NSCLC cells to PTX and CBP. Data were presented as mean ± SD. *, *P* < 0.05; **, *P* < 0.01; ***, *P* < 0.001;* ns*, no significance.

The recognition of m^6^A modification by m^6^A reader proteins is necessary for its function execution 42, 43. Thus, to determine which m^6^A reader is essential for *ABCC2* expression, we knocked down each of eight genes that have been identified as m^6^A reader in A549 and NCI-H460 cells, and strikingly found that knockdown of YTHDF1 substantially decreased mRNA expression of *ABCC2* in both NSCLC cell lines (Supplementary Figure S12A). Next, we analyzed mRNA expression of YTHDF1 in lung cancers and noncancerous lung tissues using TCGA database, and found that it was significantly up-regulated in LUADs and LUSCs compared with control subjects (Supplementary Figure S12B). These results suggest that YTHDF1 may play a potential oncogenic role in lung cancer and function as a m^6^A reader protein of *ABCC2*. To determine whether YTHDF1 can regulate mRNA stability of *ABCC2*, we first demonstrated the binding of YTHDF1 in *ABCC2* mRNA by RIP and agarose electrophoresis assay (Fig. 6A). Moreover, we found that YTHDF1 knockdown markedly down-regulated the expression of ABCC2 at both mRNA and protein levels by qRT-PCR (Fig. 6B), western blotting (Fig. 6C) and flow cytometry (Fig. 6D) assays. Particularly, YTHDF1 knockdown decreased the expression of cytomembrane-localized ABCC2 by 1.6 and 2.4 times in A549 and NCI-H460 cells, respectively (Fig. 6E). In addition, we knocked down YTHDF1 in A549 and NCI-H460 cells. After 48 h, we treated YTHDF1-knockdown cells and control cells with actinomycin D to inhibit the production of new mRNA synthesis, and monitored mRNA expression of *ABCC2* at different time points. The results showed that mRNA stability of *ABCC2* in YTHDF1-knockdown cells was significantly decreased compared with control cells (Fig. 6F). Also, we performed the luciferase reporter assay to demonstrate that, compared to the control, YTHDF1 knockdown significantly decreased the fluorescence intensity of wild-type ABCC2 reporter plasmid, but not mutant one (Fig. 6G).

Based on the above findings, we reveal the molecular mechanism by which METTL3 causes the resistance of NSCLC cells to PTX and CBP (Fig. 6H). Briefly, *ABCC2* mRNA expression was up-regulated in NSCLC cells upon PTX or CBP treatment, and further modified by METTL3-mediated m^6^A. Moreover, YTHDF1 bond with and stabilized m^6^A modified mRNA of *ABCC2* to up-regulate ABCC2 in the cytomembrane and promote the efflux of anti-cancer drugs such as PTX and CBP, thus leading to chemotherapy resistance. However, METTL3 inhibitor STM2457 could decrease the stability of *ABCC2* mRNA, resulting in down-regulated expression of ABCC2 in the cytomembrane. As a result, the efflux of PTX and CBP was reduced, thus improving the chemosensitivity of NSCLC cells.

## Discussion

Lung cancer is the most prevalent cancer with high mortality (18.0% of the total cancer deaths) in both sexes 44. Although the first-line chemotherapies responded beneficial in the beginning, the drug resistance was developed rapidly in patients with innate or acquired ability. The development of chemotherapy resistance is a complex process involving a variety of molecular alterations 45, 46. Among them, m^6^A methylation is an important layer of epigenetic modification for mRNA that mainly affects its fate, including mRNA splicing, fold, degradation and translation 24, 47. METTL3 as a major m^6^A writer to participate in all stages in the life cycle of mRNA has been demonstrated to cause the resistance to anticancer agents 48-50. However, whether METTL3 affects the resistance of NSCLC cells to PTX and CBP via m^6^A modification has not yet been fully understood.

Previous studies indicated that METTL3 substantially decreased the sensitivity of cancer cells to chemotherapy like cisplatin 51 and adriamycin 49. On the contrary, METTL3 could also sensitizes hepatocellular carcinoma cells to sorafenib by increasing mRNA stability of FOXO3 via m^6^A modification 52. In the present study, we explored the regulatory role and underlying mechanism of METTL3 involving in the chemotherapy resistance of NSCLC cells. We first found that METTL3 was highly expressed in NSCLCs, and demonstrated that METTL3 specific inhibitor STM2457 significantly inhibited the proliferation of NSCLC cells. Importantly, we found that STM2457 exhibited a synergistic anti-tumor effect with PTX or CBP. Comparing with monotherapy, a combination of STM2457 and PTX or CBP exhibited more potent *in vitro* and *in vivo* anti-tumor efficacy, indicating that targeting METTL3 enhances the sensitivity of NSCLC cells to anticancer agents such as PTX and CBP.

To elucidate the molecular mechanism by which METTL3 mediates the resistance of NSCLC cells to PTX or CBP, we found that ABCC2 was a potential target of METTL3 by a series of screening and identification. Briefly, we treated A549 and NCI-H460 cells with PTX and CBP, and found that several transporters including ABCC2 were up-regulated responsively, while their expression were significantly decreased after METTL3 inhibition. ABCC2 (also known as multi-drug resistance protein 2, MRP-2) functions as an organic anion pump in the apical epithelium of cells and participates in the transportation of anti-cancer drugs (such as cisplatin, doxorubicin, etoposide, PTX and CBP) across cell surface and intracellular organelle membrane 53-56. There is also evidence showing that the elimination of ABCC2 can retain the drug inside the cell, improving chemotherapeutic sensitivity of cancer cells 57.

Considering ABCC2 have been reported to transport PTX and CBP across the cell, we mainly focused on ABCC2 that were both up-regulated by PTX or CBP and down-regulated by STM2457. Further studies demonstrated that PTX- or CBP-induced ABCC2 was mainly localized in the cell membrane. Thus, we speculated that the drug-responsive up-regulation of ABCC2 on cytomembrane might facilitate the pumping out of anticancer drugs from the cytoplasm to extracellular, thereby leading to chemotherapeutic resistance. Next, we treated NSCLC cells with METTL3 inhibitor STM2457 or knocked down METTL3 in these cells. The results showed that STM2457 or METTL3 knockdown significantly reduced the levels of cytomembrane-localized ABCC2. These results imply that targeting METTL3 can suppress the efflux of anti-cancer drugs, improving the chemosensitivity of cancer cells.

Considering that METTL3 functions as a major m^6^A writer, we next determine whether METTL3 modulates the expression of *ABCC2* via m^6^A modification. We first found a putative m^6^A methylation site located in the CDS of *ABCC2* mRNA by analyzing several online databases, and demonstrated that STM2457 or METTL3 knockdown significantly decreased the levels of m^6^A methylation at this putative site using MeRIP-qPCR assay. Moreover, we used the luciferase reporter assay to further verify that METTL3 regulated the mRNA expression of *ABCC2* via a m^6^A-dependent manner. To comprehend more about how m^6^A modification modulates mRNA stability of *ABCC2*, we knocked down all well-known m^6^A reader proteins in NSCLC cells to figure out which one recognized this site and participated in the regulation of its mRNA stability. Consistently, we found that knocking down YTHDF1 in NSCLC cells substantially reduced the expression of ABCC2 at both mRNA and protein levels. YTHDF1 as an important m^6^A reader plays critical roles in different types of human cancers, which are involved in regulating DNA damage repair, proliferation, metastasis, immunity and chemoresistance 58-60. Mechanistically, YTHDF1 is able to recognize and stabilize the target mRNAs 52, 61. Also, it can recruit the translation machinery to target mRNAs, facilitating protein translation 59, 62-64. In addition, we also demonstrated that YTHDF1 knockdown decreased mRNA stability of *ABCC2* and the firefly luciferase intensity of ABCC2 reporter plasmid containing wild-type m^6^A site, but not mutant one. These results indicate that METTL3-mediated m^6^A modification increases mRNA stability of *ABCC2* in an YTHDF1-dependent manner. In addition to YTHDF1, we do not rule out that other m^6^A readers may also be involved in regulating mRNA stability of *ABCC2*, thereby contributing to the resistance of NSCLC cells to chemotherapeutic agents, such as PTX and CBP.

## Conclusions

In summary, we uncover an important role of METTL3-mediated m^6^A modification in promoting PTX and CBP resistance in NSCLC. In this study, we identify that ABCC2 is a direct target of METTL3, and find that PTX and CBP can up-regulate ABCC2 expression in cytomembrane, which may contribute to the resistance of NSCLC cells to PTX or CBP. Targeting METTL3 down-regulates ABCC2 expression in cytomembrane in an m^6^A-YTHDF1-dependent manner. This will increase the intracellular concentration of PTX and CBP by preventing their efflux, thereby improving the chemosensitivity of NSCLC. From the macro perspective, the present study indicates that METTL3 may be a potential therapeutic target in NSCLC, and reveals that the METTL3/ABCC2 axis serves as a critical regulator in PTX- and CBP-resistant NSCLCs. More importantly, it will provide a new and safe strategy to re-sensitize chemotherapy-resistant NSCLCs to anti-cancer drugs.

## Supplementary Material

Supplementary figures and tables.

## Abbreviations

ABCC2: ATP binding cassette subfamily C member 2
ALKBH5: AlkB homolog H5
CBP: carboplatin
CDS: coding sequence
FOXO3: forkhead box O3
FTO: fat mass and obesity associated
H&E: hematoxylin and eosin
IC_50_: half maximal inhibitory concentration
IF: immunofluorescence
IHC: immunohistochemistry
LUAD: lung adenocarcinoma
LUSC: lung squamous cell carcinoma
m^6^A: n^6^-methyladenosine
METTL3: methyltransferase-like 3
METTL14: methyltransferase-like 14
MeRIP-qPCR: methylated RNA immunoprecipitation and qPCR
MDR: multidrug resistance
mRNA: messenger RNA
ncRNAs: non-coding RNAs
NSCLC: non-small cell lung cancer
PD-L1: programmed cell death ligand 1
PTX: paclitaxel
qRT-PCR: quantitative real time -PCR
RBM15: RNA binding motif protein 15
siRNAs: short interfering RNAs
SPF: specific pathogen-free
WTAP: wilms tumor 1-associated protein
YTHDF1: YTH n^6^-methyladenosine RNA binding protein F1
γH2AX: phosphorylation of the histone H2A variant

## References

[1] Rodak O, Peris-Díaz MD, Olbromski M, Podhorska-Okołów M, Dzięgiel P (2021). Current landscape of non-small cell lung cancer: epidemiology, histological classification, targeted therapies, and immunotherapy. Cancers.

[2] Duma N, Santana-Davila R, Molina JR (2019). Non-Small Cell Lung Cancer: epidemiology, screening, diagnosis, and treatment. Mayo Clin Proc.

[3] Fois SS, Paliogiannis P, Zinellu A, Fois AG, Cossu A, Palmieri G (2021). Molecular epidemiology of the main druggable genetic alterations in non-small cell lung cancer. Int J Mol Sci.

[4] Ganti AK, Klein AB, Cotarla I, Seal B, Chou E (2021). Update of incidence, prevalence, survival, and Iinitial treatment in patients with non-small cell lung cancer in the US. JAMA Oncol.

[5] Yang Y, Qian ZY, Feng MY, Liao WT, Wu QJ, Wen F (2022). Study on the prognosis, immune and drug resistance of m6A-related genes in lung cancer. BMC Bioinformatics.

[6] Doroshow DB, Sanmamed MF, Hastings K, Politi K, Rimm DL, Chen L (2019). Immunotherapy in non-small cell lung cancer: facts and hopes. Clin Cancer Res.

[7] Cowper PA, Feng L, Kosinski AS, Tong BC, Habib RH, Putnam JB (2021). Initial and longitudinal cost of surgical resection for lung cancer. Ann Thorac Surg.

[8] Bade BC, Dela Cruz CS (2020). Lung cancer 2020. Clin Chest Med.

[9] Imyanitov EN, Iyevleva AG, Levchenko EV (2021). Molecular testing and targeted therapy for non-small cell lung cancer: current status and perspectives. Crit Rev Oncol Hematol.

[10] Vinod SK, Hau E (2020). Radiotherapy treatment for lung cancer: Current status and future directions. Respirology.

[11] Xie Y, Feng SL, He F, Yan PY, Yao XJ, Fan XX (2022). Down-regulating Nrf2 by tangeretin reverses multiple drug resistance to both chemotherapy and EGFR tyrosine kinase inhibitors in lung cancer. Pharmacol Res.

[12] Miao YQ, Chen MS, Zhou X, Guo LM, Zhu JJ, Wang R (2021). Chitosan oligosaccharide modified liposomes enhance lung cancer delivery of paclitaxel. Acta Pharmacol Sin.

[13] Lv PP, Man SL, Xie L, Ma L, Gao WY (2021). Pathogenesis and therapeutic strategy in platinum resistance lung cancer. Biochim Biophys Acta Rev Cancer.

[14] Weaver BA, Bement W (2014). How taxol/paclitaxel kills cancer cells. Mol Biol Cell.

[15] Roos WP, Kaina B (2013). DNA damage-induced cell death: from specific DNA lesions to the DNA damage response and apoptosis. Cancer Lett.

[16] Tian DM, Tang JS, Geng XR, Li Q, Wang FF, Zhao HD (2020). Targeting UHRF1-dependent DNA repair selectively sensitizes KRAS mutant lung cancer to chemotherapy. Cancer Lett.

[17] Ween MP, Armstrong MA, Oehler MK, Ricciardelli C (2015). The role of ABC transporters in ovarian cancer progression and chemoresistance. Crit Rev Oncol Hematol.

[18] Liu ZQ, Zou HJ, Dang Q, Xu H, Liu L, Zhang YY (2022). Biological and pharmacological roles of m6A modifications in cancer drug resistance. Mol Cancer.

[19] Gottesman MM (2002). Mechanisms of cancer drug resistance. Int J Mol Sci.

[20] Kathawala RJ, Gupta P, Ashby CR, Chen ZS (2015). The modulation of ABC transporter-mediated multidrug resistance in cancer: a review of the past decade. Drug Resist Updat.

[21] Huang HL, Weng HY, Chen JJ (2020). M6A modification in coding and non-coding RNAs: roles and therapeutic implications in cancer. Cancer Cell.

[22] Liu XY, Feng MX, Hao XD, Gao ZH, Wu ZX, Wang YL (2023). m6A methylation regulates hypoxia-induced pancreatic cancer glycolytic metabolism through ALKBH5-HDAC4-HIF1α positive feedback loop. Oncogene.

[23] Liu SP, Li QJ, Li GH, Zhang Q, Zhuo LJ, Han XM (2020). The mechanism of m6A methyltransferase METTL3-mediated autophagy in reversing gefitinib resistance in NSCLC cells by β-elemene. Cell Death Dis.

[24] Sendinc E, Shi Y (2023). RNA m6A methylation across the transcriptome. Mol cell.

[25] Liu L, Wu Y, Li Q, Liang JF, He QT, Zhao LD (2020). METTL3 promotes tumorigenesis and metastasis through BMI1 m6A methylation in oral squamous cell carcinoma. Mol Ther.

[26] Peng W, Li J, Chen RR, Gu Q, Yang P, Qian WW (2019). Upregulated METTL3 promotes metastasis of colorectal cancer via miR-1246/SPRED2/MAPK signaling pathway. J Exp Clin Cancer Res.

[27] Dang H, Sui MJ, He QY, Xie JY, Liu Y, Hou P (2023). Pin1 inhibitor API-1 sensitizes BRAF-mutant thyroid cancers to BRAF inhibitors by attenuating HER3-mediated feedback activation of MAPK/ERK and PI3K/AKT pathways. Int J Biol Macromol.

[28] Shi J, Qu YP, Li XR, Sui F, Yao DM, Yang Q (2016). Increased expression of EHF via gene amplification contributes to the activation of HER family signaling and associates with poor survival in gastric cancer. Cell Death Dis.

[29] Ma SR, Wang N, Liu R, Zhang R, Dang H, Wang YB (2021). ZIP10 is a negative determinant for anti-tumor effect of mannose in thyroid cancer by activating phosphate mannose isomerase. J Exp Clin Cancer Res.

[30] Li T, Hu PS, Zuo ZX, Lin JF, Li XY, Wu QN (2019). METTL3 facilitates tumor progression via an m6A-IGF2BP2-dependent mechanism in colorectal carcinoma. Mol Cancer.

[31] Shi LX, Ma R, Lu R, Xu Q, Zhu ZF, Wang L (2008). Reversal effect of tyroservatide (YSV) tripeptide on multi-drug resistance in resistant human hepatocellular carcinoma cell line BEL-7402/5-FU. Cancer Lett.

[32] Smith ER, Wang JQ, Yang DH, Xu XX (2022). Paclitaxel resistance related to nuclear envelope structural sturdiness. Drug Resist Updat.

[33] Zhao YN, Peng HT, Liang LM, Li Y, Hu XC, Wang BY (2022). Polarity protein Par3 sensitizes breast cancer to paclitaxel by promoting cell cycle arrest. Breast Cancer Res.

[34] Jung Y, Lippard SJ (2007). Direct cellular responses to platinum-induced DNA damage. Chem Rev.

[35] He J, Fortunati E, Liu DX, Li Y (2021). Pleiotropic roles of ABC transporters in breast cancer. Int J Mol Sci.

[36] Locher KP (2016). Mechanistic diversity in ATP-binding cassette (ABC) transporters. Nat Struct Mol Biol.

[37] Theile D, Wizgall P (2021). Acquired ABC-transporter overexpression in cancer cells: transcriptional induction or Darwinian selection?. Naunyn Schmiedebergs Arch Pharmacol.

[38] Wang X, Zhang HY, Chen XZ (2019). Drug resistance and combating drug resistance in cancer. Cancer Drug Resist.

[39] Bukowski K, Kciuk M, Kontek R (2020). Mechanisms of multidrug resistance in cancer chemotherapy. Int J Mol Sci.

[40] Huisman MT, Chhatta AA, van Tellingen O, Beijnen JH, Schinkel AH (2005). MRP2 (ABCC2) transports taxanes and confers paclitaxel resistance and both processes are stimulated by probenecid. Int J Cancer.

[41] Liedert B, Materna V, Schadendorf D, Thomale J, Lage H (2003). Overexpression of cMOAT (MRP2/ABCC2) is associated with decreased formation of platinum-DNA adducts and decreased G2-arrest in melanoma cells resistant to cisplatin. J Invest Dermatol.

[42] Luo JY, Liu H, Luan SY, He CS, Li ZY (2018). Aberrant regulation of mRNA m6A modification in cancer development. Int J Mol Sci.

[43] He LE, Li HY, Wu AQ, Peng YL, Shu G, Yin G (2019). Functions of N6-methyladenosine and its role in cancer. Mol Cancer.

[44] Sung H, Ferlay J, Siegel RL, Laversanne M, Soerjomataram I, Jemal A (2021). Global cancer statistics 2020: GLOBOCAN Estimates of incidence and mortality worldwide for 36 cancers in 185 countries. CA-Cancer J Clin.

[45] Chatterjee N, Bivona TG (2019). Polytherapy and targeted cancer drug resistance. Trends Cancer.

[46] Ward RA, Fawell S, Floc'h N, Flemington V, McKerrecher D, Smith PD (2020). Challenges and opportunities in cancer drug resistance. Chem Rev.

[47] Sun T, Wu RY, Ming L (2019). The role of m6A RNA methylation in cancer. Biomed Pharmacother.

[48] Pan SL, Deng YY, Fu J, Zhang YH, Zhang ZJ, Qin XJ (2022). N6-methyladenosine upregulates miR-181d-5p in exosomes derived from cancer-associated fibroblasts to inhibit 5-FU sensitivity by targeting NCALD in colorectal cancer. Int J Oncol.

[49] Pan XP, Hong XL, Li SM, Meng P, Xiao F (2021). METTL3 promotes adriamycin resistance in MCF-7 breast cancer cells by accelerating pri-microRNA-221-3p maturation in a m6A-dependent manner. Exp Mol Med.

[50] Sun YQ, Shen WT, Hu SL, Lyu Q, Wang QY, Wei T (2023). METTL3 promotes chemoresistance in small cell lung cancer by inducing mitophagy. J Exp Clin Cancer Res.

[51] Zhang Y, Qiu JG, Jia XY, Ke Y, Zhang MK, Stieg D (2023). METTL3-mediated N6-methyladenosine modification and HDAC5/YY1 promote IFFO1 downregulation in tumor development and chemo-resistance. Cancer Lett.

[52] Lin ZY, Niu Y, Wan A, Chen DS, Liang H, Chen XJ (2020). RNA m6A methylation regulates sorafenib resistance in liver cancer through FOXO3-mediated autophagy. EMBO J.

[53] Gao B, Lu Y, Nieuweboer AJM, Xu HM, Beesley J, Boere I (2018). Genome-wide association study of paclitaxel and carboplatin disposition in women with epithelial ovarian cancer. Sci Rep.

[54] Němcová-Fürstová V, Kopperová D, Balušíková K, Ehrlichová M, Brynychová V, Václavíková R (2016). Characterization of acquired paclitaxel resistance of breast cancer cells and involvement of ABC transporters. Toxicol Appl Pharmacol.

[55] Xie BW, Wang SY, Jiang N, Li JJ (2019). Cyclin B1/CDK1-regulated mitochondrial bioenergetics in cell cycle progression and tumor resistance. Cancer Lett.

[56] Lambrechts S, Lambrechts D, Despierre E, Van Nieuwenhuysen E, Smeets D, Debruyne PR (2015). Genetic variability in drug transport, metabolism or DNA repair affecting toxicity of chemotherapy in ovarian cancer. BMC Pharmacol Toxicol.

[57] Marsh S, Paul J, King CR, Gifford G, McLeod HL, Brown R (2007). Pharmacogenetic assessment of toxicity and outcome after platinum plus taxane chemotherapy in ovarian cancer: the scottish randomised trial in ovarian cancer. J Clin Oncol.

[58] Han DL, Liu J, Chen CY, Dong LH, Liu Y, Chang RB (2019). Anti-tumour immunity controlled through mRNA m6A methylation and YTHDF1 in dendritic cells. Nature.

[59] Wang SY, Gao SS, Zeng Y, Zhu L, Mo YL, Wong CC (2022). N6-methyladenosine reader YTHDF1 promotes ARHGEF2 translation and RhoA signaling in colorectal cancer. Gastroenterology.

[60] Sun Y, Dong D, Xia YH, Hao LY, Wang W, Zhao CH (2022). YTHDF1 promotes breast cancer cell growth, DNA damage repair and chemoresistance. Cell Death Dis.

[61] Tang WM, Zhao YL, Zhang H, Peng Y, Rui ZL (2022). METTL3 enhances NSD2 mRNA stability to reduce renal impairment and interstitial fibrosis in mice with diabetic nephropathy. BMC Nephrol.

[62] Wang X, Zhao BS, Roundtree Ian A, Lu ZK, Han DL, Ma HH (2015). N6-methyladenosine modulates messenger RNA translation efficiency. Cell.

[63] Yi P, Zou DL, Yu J, Yu JH, Wang F, Rao S (2020). The m6A reader YTHDF1 promotes ovarian cancer progression via augmenting EIF3C translation. Nucleic Acids Res.

[64] Jiang XL, Liu BY, Nie Z, Duan LC, Xiong QX, Jin ZX (2021). The role of m6A modification in the biological functions and diseases. Signal Transduct Target Ther.

