# Supplementary material for: Targeting METTL3 enhances the chemosensitivity of non-small cell lung cancer cells by decreasing ABCC2 expression in an m6A-YTHDF1-dependent manner
Source: Int J Biol Sci. 2024 Sep 3;20(12):4750–66. doi: 10.7150/ijbs.97425 (PMC11414383; doi:10.7150/ijbs.97425)
Supplement: Supplementary file 1 — Supplementary figures and tables. [file ijbsv20p4750s1.pdf]

# Supplementary Figures

# Targeting METTL3 enhances the chemosensitivity of non-small cell lung cancer cells by decreasing ABCC2 expression in an $m^6A$-YTHDF1-dependent manner

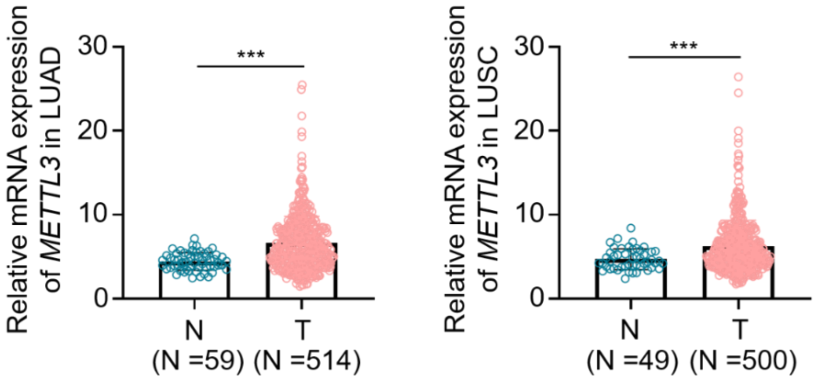


**Figure S1. *METTL3* mRNA expression in lung cancers**

The mRNA expression of METTL3 was analyzed in LUADs and LUSCs and noncancerous lung tissues (the data from the TCGA database). N: noncancerous lung tissue, T: tumor tissue. Data were presented as mean ± SD. ***, $P < 0.001$.

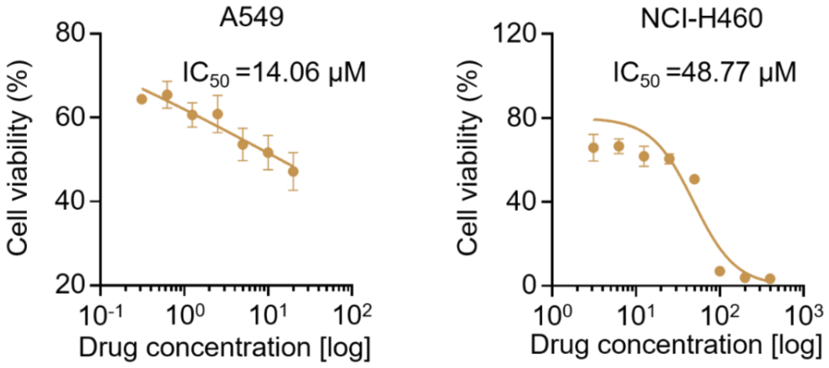


**Figure S2. The $IC_{50}$ values of STM2457 in NSCLC cells.**

A549 and NCI-H460 cells were treated with indicated concentrations of STM2457 for 48 h. MTT assay was then performed to evaluated cell viability, and Reed-Muench method was used to calculated the $IC_{50}$ values. The data were presented as the mean ± SD.

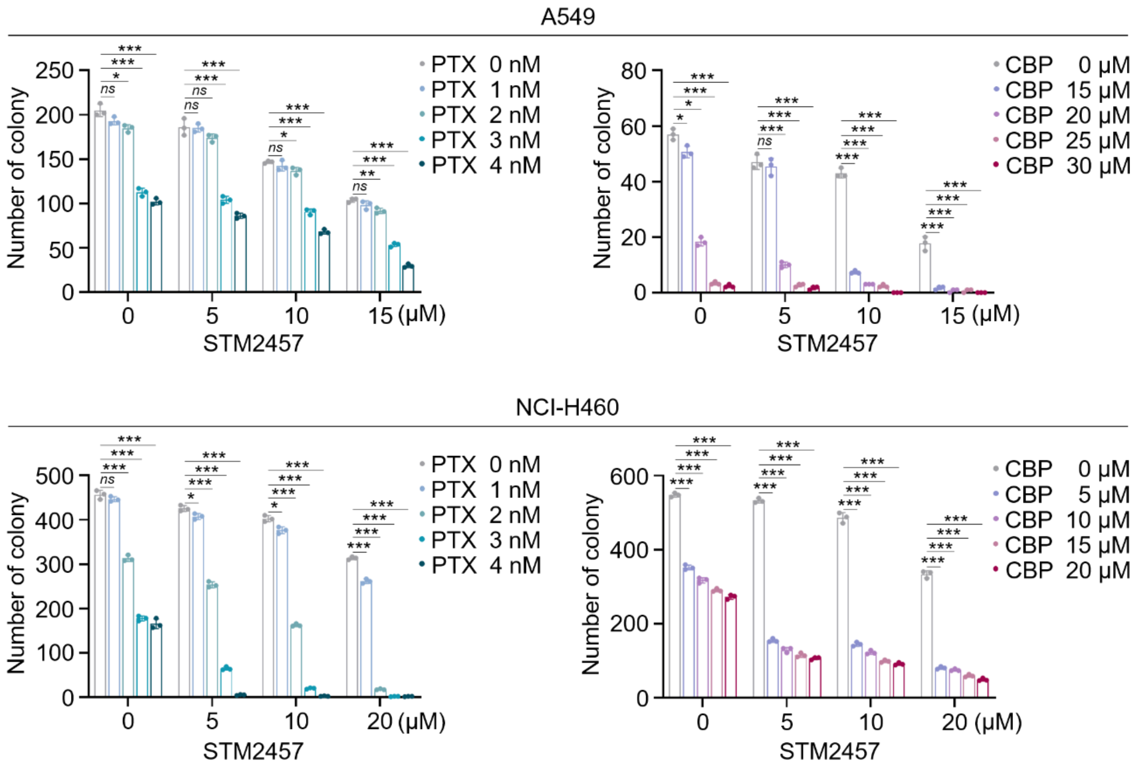


**Figure S3. The statistical analysis of clones**

A549 and NCI-H460 cells were treated with indicated PTX or CBP individually or in combination with different dose of STM2457, and their effect on cell colony formation ability was then evaluated. The number of clones was statistically analyzed. The data were presented as the mean ± SD. *, $P$ <0.05; **, $P$ <0.01; ***, $P$ <0.001; *ns*, no significance.

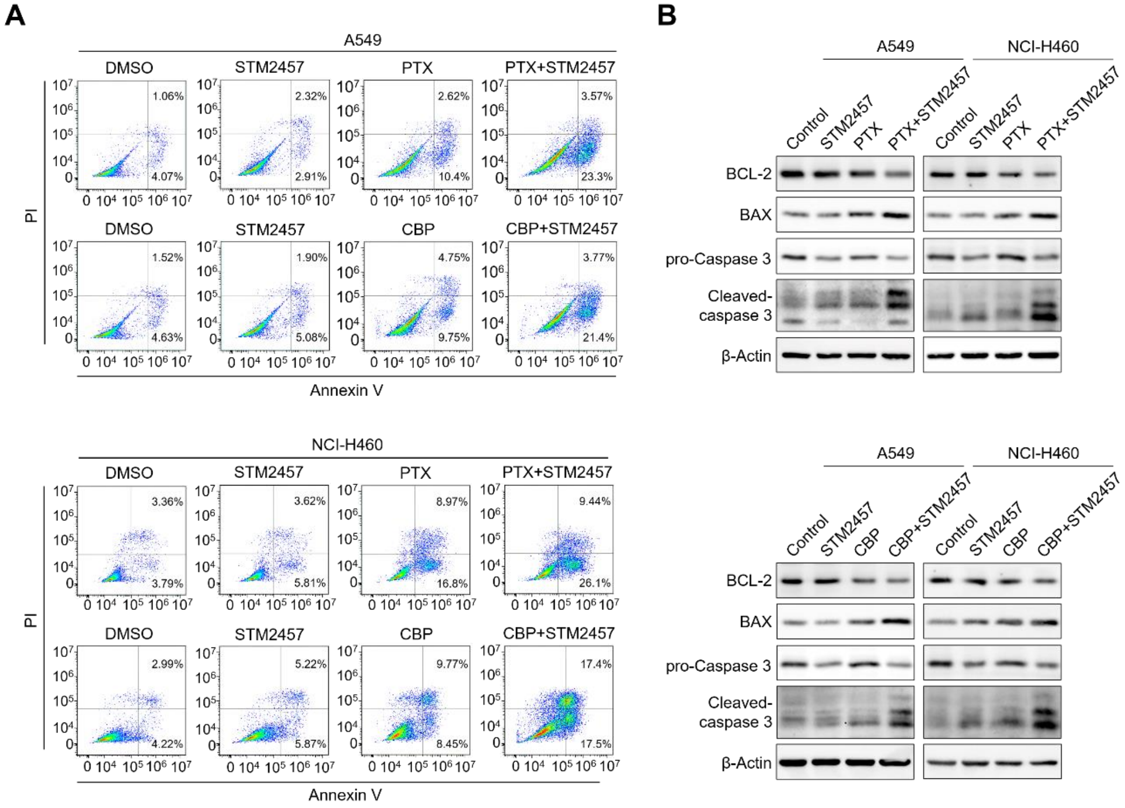


**Figure S4. The effect of STM2457, individually or in combination with PTX or CBP, on the apoptosis of NSCLC cells**

A549 and NCI-H460 cells were treated with 5 μM or 20 μM STM2457 individually or in combination with 5 nM PTX or 30 μM CBP for 48 h. (**A**) Cell apoptosis was then assessed by flow cytometry. (**B**) The levels of apoptosis-related markers were detected by western blotting analysis. β-Actin was used as a loading control.

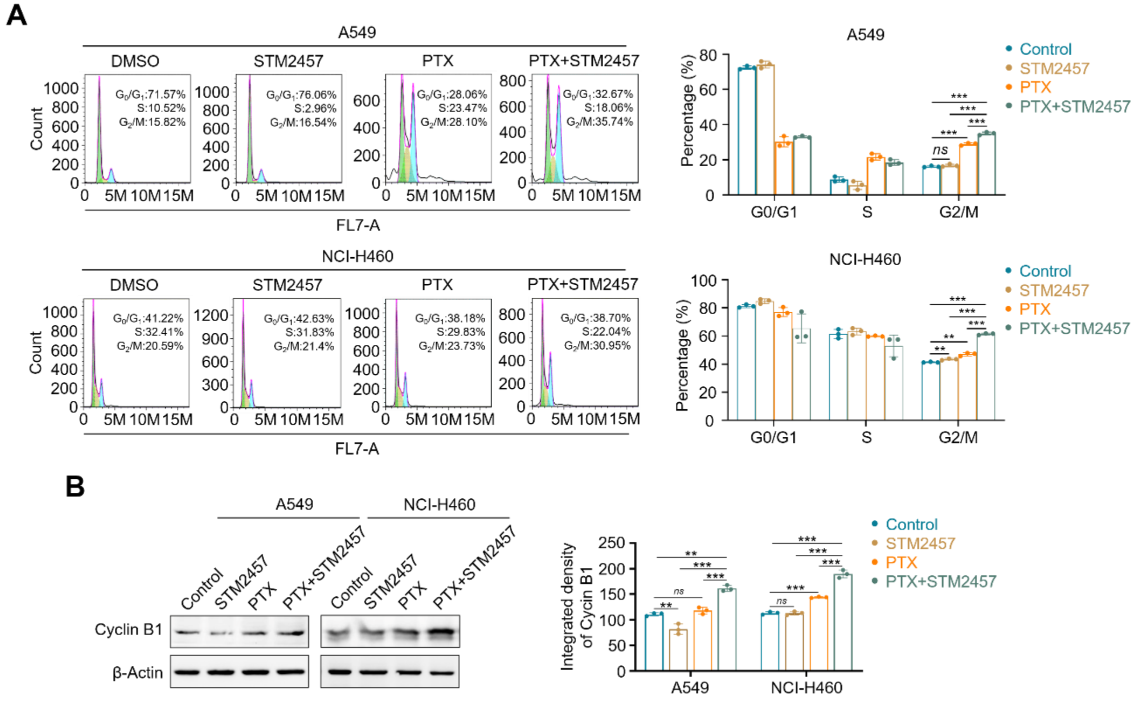


**Figure S5. The effect of STM2457, individually or in combination with PTX or CBP, on cell cycle distribution of NSCLC cells**

**(A)** A549 and NCI-H460 cells were treated with 5 μM or 20 μM STM2457 individually or in combination with 5 nM PTX or 30 μM CBP. After a 24-h treatment, cell cycle was assessed by flow cytometry. Representative flow cytometric histograms were shown in the left panel, and the percentage of each cell cycle phase was indicated in the right panel. **(B)** The levels of cyclin B1 were detected by western blotting analysis and integrated density was analyzed statistically. The representative images of cyclin B1 were shown in the left panels, and statistical analysis of integrated density of cyclin B1 was shown in the right panel. β-Actin was used as a loading control. Data were presented as mean ± SD. *, $P < 0.05$; **, $P < 0.01$; ***, $P < 0.001$; *ns*, no significance.

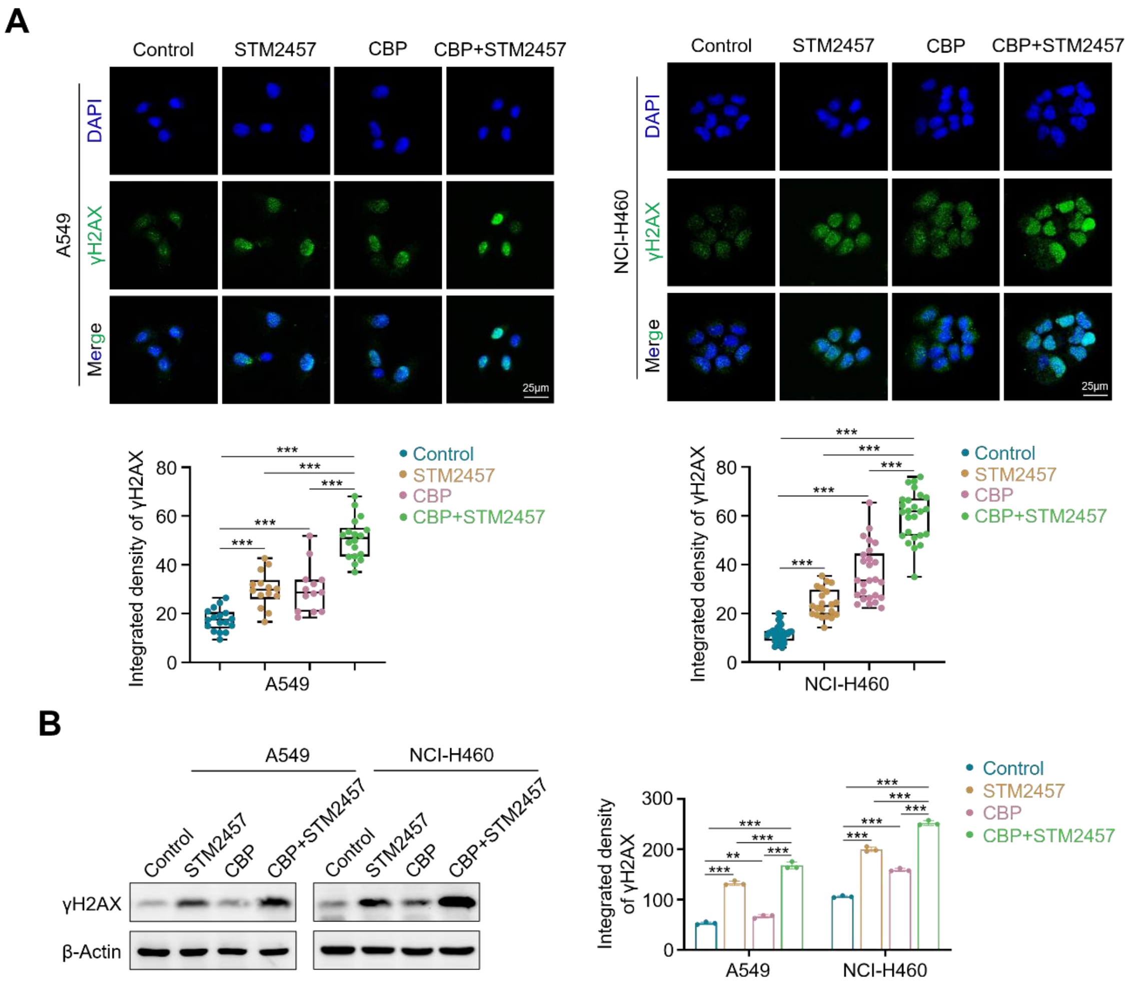


**Figure S6. The effect of STM2457, individually or in combination with PTX or CBP, on DNA damage of NSCLC cells**

**(A)** A549 and NCI-H460 cells were treated with 5 μM or 20 μM STM2457 individually or in combination with 5 nM PTX or 30 μM CBP. After a 48-h treatment, γH2AX foci was detected by immunofluorescence assay. The representative immunofluorescence images of γH2AX were shown in the upper panels, and statistical analysis of integrated density of γH2AX was shown in the lower panels. Blue color indicates the staining of nuclei, and

green color indicates the staining of γH2AX. Scale bars, 25 μm. **(B)** A549 and NCI-H460 cells were treated with the same conditions as above for 48 h. The protein expression of γH2AX was detected by western blotting analysis. The representative images of γH2AX were shown in the left panel, and statistical analysis of integrated density of γH2AX was shown in the right panel. β-Actin was used as a loading control.

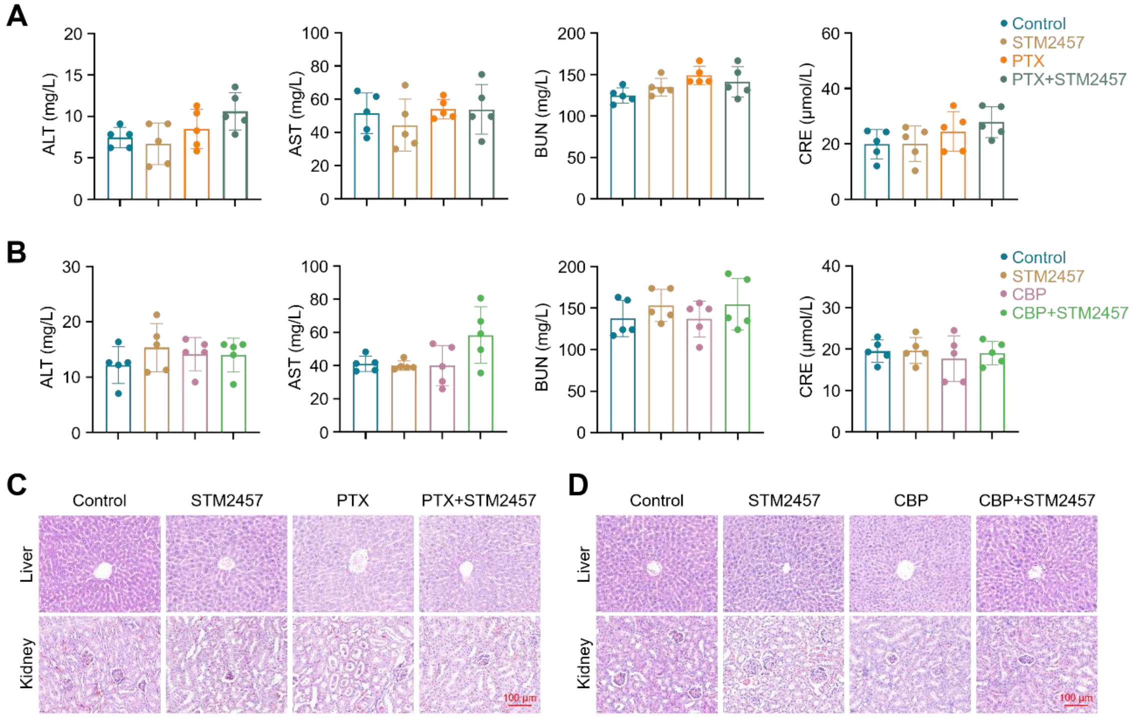


**Figure S7. Safety evaluation of drugs in nude mice**

**(A-B)** Tumor-bearing nude mice were grouped randomly (n=5/group) and treated with STM2457 (30 mg/kg, once a day) and PTX (3 ng/kg, once every two days) or CBP (30 mg/kg, once every two days), individually or in combination, for 2 weeks. The levels of alanine transaminase (ALT), aspartate aminotransferase (AST), blood urea nitrogen (BUN) and serum creatinine (CRE) were detected by corresponding kits. **(C-D)** Representative

H&E staining of kidney and liver sections from the indicated groups. Scale bar, 100 μm. Data were presented as mean ± SD.

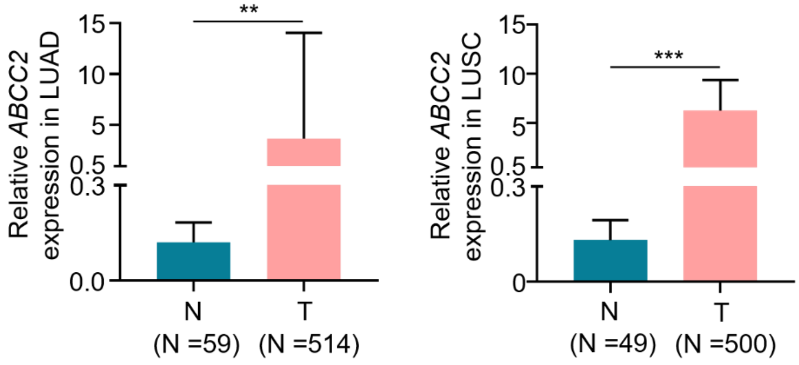


**Figure S8. *ABCC2* mRNA expression in lung cancers**

*ABCC2* mRNA expression was analyzed in LUADs and LUSCs in comparison with unpaired noncancerous lung tissues (the data from the TCGA database). N: noncancerous lung tissue, T: tumor tissue. Data were presented as mean ± SD. **, $P < 0.01$; ***, $P < 0.001$.

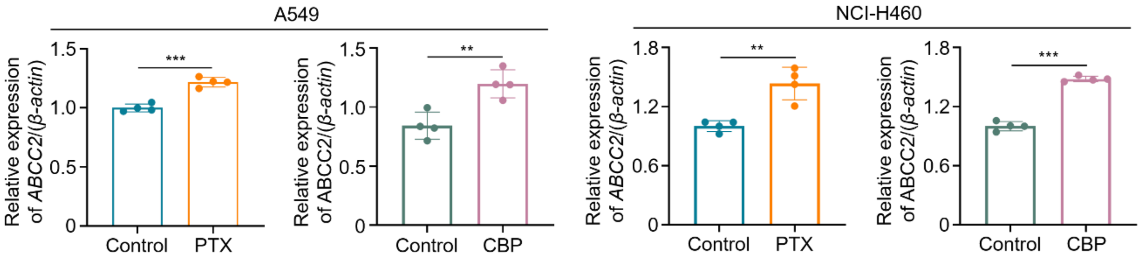


**Figure S9. The effect of PTX or CBP on mRNA expression of ABCC2**

A549 cells were treated 3 nM PTX or 20 μM CBP and NCI-H460 cells were treated 5 nM PTX or 30 μM CBP for 24 h, the mRNA expression of *ABCC2* was measured by qRT-PCR

assay. *β-actin* was used as the normalization control. Data were presented as mean ± SD. *, $P < 0.05$; **, $P < 0.01$; ***, $P < 0.001$.

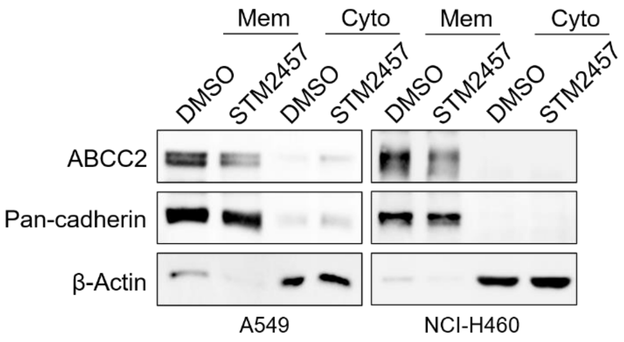


**Figure S10. The protein expression of ABCC2 in cytomembrane and cytosol**

A549 and NCI-H460 cells were treated with 5 μM or 20 μM STM2457, respectively. The proteins in membrane and cytosol were extracted separately, and western blotting analysis was then performed to detect the production of ABCC2 in cytomembrane and cytosol. β-Actin was used as a loading control of cytoplasm proteins, and pan-cadherin was used a loading control of cytomembrane proteins. Mem, Membrane; Cyto, Cytosol.

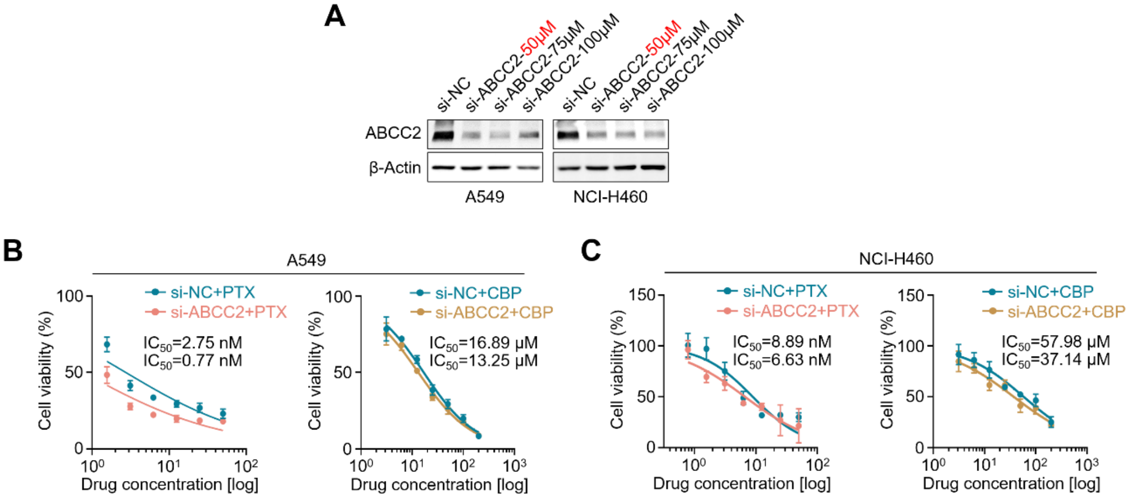


**Figure S11. The regulatory effect of ABCC2 on the sensitivity of PTX and CBP**

**(A)** A549 and NCI-H460 cells were treated with different doses of siRNA targeting ABCC2, and the protein levels of ABCC2 were measured by western blotting analysis. β-Actin was used as a loading control. (**B**-**C**) ABCC2-knockdown A549 and NCI-H460 cells and their control cells were treated with the indicated doses of PTX or CBP for 48 h. MTT assay was then performed to evaluated cell viability, and Reed-Muench method was used to calculated the $IC_{50}$ values. The data were presented as the mean ± SD.

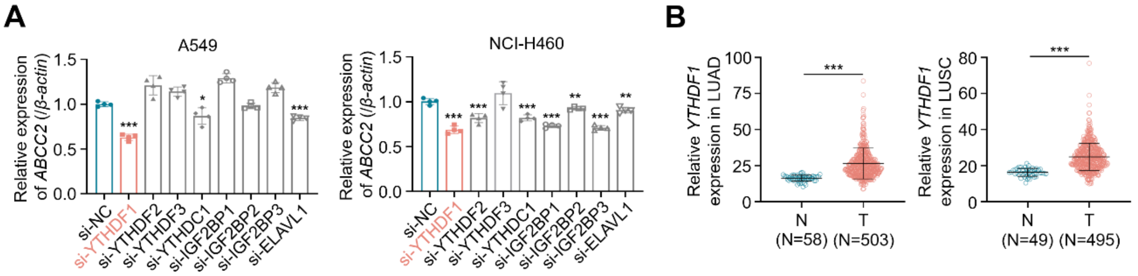


**Figure S12. The regulatory effect of YTHDF1 on mRNA expression of *ABCC2***

**(A)** The indicated $m^6A$ reader proteins were knocked down in A549 and NCI-H460 cells,

and the mRNA expression of *ABCC2* was measured by qRT-PCR. *β-actin* was used as a normalization control. **(B)** *YTHDF1* mRNA expression was analyzed in LUAD and LUSC tissues in comparison with unpaired normal lung tissues (the data from the TCGA database). N: noncancerous lung tissue, T: tumor tissue. Data were presented as mean ± SD. *, $P < 0.05$; **, $P < 0.01$; ***, $P < 0.001$.

## Supplementary Tables

### Supplementary Table S1: Sequence of siRNAs

| siRNAs | Sequence (5’-3’) |
|---|---|
| si-NC | 5'-UUCUCCGAACGUGUCACGUTT-3’ |
| si-METTL3#1 | 5'-CAAGTATGTTCACTATGAA-3’ |
| si-METTL3#2 | 5'-GACTGCTCTTTCCTTAATA-3’ |
| si-YTHDF1#1 | 5'-ACGGCAGAGTCGAAACAAA-3’ |
| si-YTHDF1#2 | 5′-CCTCCACCCATAAAGCATA-3′ |
| si-YTHDF2 | 5′-AAGGACGTTCCCAATAGCCAA-3′ |
| si-YTHDF3 | 5′-AGAUGGUGUAUUUAGUCAACC-3′ |
| si-YTHDC1 | 5’-GAAGUGGAUAGACGUGCAATT-3’ |
| si-IGF2BP1 | 5’-GGCCCAUAAUAACUUUGUATT-3’ |
| si-IGF2BP2 | 5’-CATGCCGCATGATTCTTGA-3’ |
| si-IGF2BP3 | 5’-GCAGGAAUUGACGCUGUAUTT-3’ |
| si-ELAVL1 | 5′-AAGAGGCAAUUACCAGUUUCA-3′ |
| si-ABCC2 | 5′-CCATAGCTTCATTCCTGAGTA-3′ |

### Supplementary Table S2: The primers of qRT-PCR used in this study

| Genes | Primer sequences |
|---|---|
| β-actin | Forward primer (5’-3’), CCTTGCACATGCCGGAG; |
| | Reverse primer (3’-5’), GCACAGAGCCTCGCCTT. |
| METTL3 | Forward primer (5’-3’), CAAGCTGCACTTCAGACGAA; |
| | Reverse primer (3’-5’), GCTTGGCGTGTGGTCTTT. |
| ABCC2 | Forward primer (5’-3’), CCCTGCTGTTCGATATACCAATC; |
| | Reverse primer (3’-5’), TCGAGAGAATCCAGAATAGGGAC. |
| 18S rRNA | Forward primer (5’-3’), CAGCCACCCGAGATTGAGCA; |
| | Reverse primer (3’-5’), TAGTAGCGACGGGCGGTGTG. |
| YTHDF1 | Forward primer (5’-3’), ACCTGTCCAGCTATTACCCG; |
| | Reverse primer (3’-5’), TGGTGAGGTATGGAATCGGAG. |

| | | |
|---|---|---|
| YTHDF2 | Forward primer (5'-3'), TAGCCAACTGCGACACATTC; | |
| | Reverse primer (3'-5'), CACGACCTTGACGTTCCTTT. | |
| YTHDF3 | Forward primer (5'-3'), GGTGTATTTAGTCAACCTGGGG; | |
| | Reverse primer (3'-5'), AAGAGAACTAGGTGGATAGCCAT. | |
| YTHDC1 | Forward primer (5'-3'), AAGGAGGGCCAAATCTCCTA; | |
| | Reverse primer (3'-5'), CAGTGTTGTTCCCTTGCTCA. | |
| IGF2BP1 | Forward primer (5'-3'), AGAATGATGTGGCTGCCATGA; | |
| | Reverse primer (3'-5'), AAAGGAGCTATAGGGAGCAGC. | |
| IGF2BP2 | Forward primer (5'-3'), AGCTAAGCGGGCATCAGTTTG; | |
| | Reverse primer (3'-5'), CCGCAGCGGGAAATCAATCT. | |
| IGF2BP3 | Forward primer (5'-3'), TATATCGGAAACCTCAGCGAGA; | |
| | Reverse primer (3'-5'), GGACCGAGTGCTCAACTTCT. | |
| ELAVL1 | Forward primer (5'-3'), TGTTCTCTCGGTTTGGGCGGAT; | |
| | Reverse primer (3'-5'), TCTTCTGCCTCCGACCGTTTGT. | |
| ABCA1 | Forward primer (5'-3'), GCAAGGCTACCAATTACATTTG; | |
| | Reverse primer (3'-5'), GGTCAGAAACATCACCTCCTG. | |
| ABCA2 | Forward primer (5'-3'), GCCCAGGTCTGGCTCAACATCTC; | |
| | Reverse primer (3'-5'), CTCACCTTGGACATGAACTGGAT. | |
| ABCA3 | Forward primer (5'-3'), CTTGACAGTCGCAGAGCACCTT; | |
| | Reverse primer (3'-5'), CTCCGTGAGTTCCACTTGTCCT. | |
| ABCA8 | Forward primer (5'-3'), GGCCCTTTTCTTGGCACTTG; | |
| | Reverse primer (3'-5'), CAGGCCGGTGAGGAAAGATT. | |
| ABCB1 | Forward primer (5'-3'), TGCTCAGACAGGATGTGAGTTG; | |
| | Reverse primer (3'-5'), AATTACAGCAAGCCTGGAACC. | |
| ABCB4 | Forward primer (5'-3'), GCATCAGCAGCAAACAAAAA; | |
| | Reverse primer (3'-5'), GCAGCGACAAGGAAAAGTTC. | |
| ABCB5 | Forward primer (5'-3'), TTTCTCCGCCAGCATTCCAT; | |
| | Reverse primer (3'-5'), GCTGAGGAATCCACCCAATCT. | |
| ABCB8 | Forward primer (5'-3'), CTGTCAGGTACTCTGATGGCT; | |
| | Reverse primer (3'-5'), TCCATCTGGGAGCTAGGGG. | |
| ABCB11 | Forward primer (5'-3'), ACTAGATGAAGCCACTTCTGCCTTA; | |

| | | |
|---|---|
| | Reverse primer (3'-5'), TGCACCGTCTTTTCACTTTCTGT. |
| ABCC1 | Forward primer (5'-3'), CTCTATCTCTCCCGACATGACC; |
| | Reverse primer (3'-5'), AGCAGACGATCCACAGCAAAA. |
| ABCC3 | Forward primer (5'-3'), AGCTCGGCTCCAAGTTCTG; |
| | Reverse primer (3'-5'), GACCCACAGGTAGATGCAGG. |
| ABCC4 | Forward primer (5'-3'), AGCTGAGAATGACGCACAGAA; |
| | Reverse primer (3'-5'), ATATGGGCTGGATTACTTTGGC. |
| ABCC5 | Forward primer (5'-3'), AGTCCTGGGTATAGAAGTGTGAG; |
| | Reverse primer (3'-5'), ATTCCAACGGTCGAGTTCTCC. |
| ABCC6 | Forward primer (5'-3'), CTGGACGAGGCTACTGCTG; |
| | Reverse primer (3'-5'), TTGTCCATGACCAGAACCC. |
| ABCC10 | Forward primer (5'-3'), CGGCCTGCTCTATGCTCTG; |
| | Reverse primer (3'-5'), CCCCGTGCCTGAAGTGTTA. |
| ABCC11 | Forward primer (5'-3'), CCTACTTCATTATTGGATACACTGC; |
| | Reverse primer (3'-5'), CTTGTCATGAATACCGCCAG. |
| ABCE1 | Forward primer (5'-3'), CACAGGTTGCCTATCCCTCG; |
| | Reverse primer (3'-5'), AAATAAGTCAAAATCTCCTGCCAGTCAG. |
| ABCG1 | Forward primer (5'-3'), GTCTGAACTGCCCAACCTACCAC; |
| | Reverse primer (3'-5'), CCGACTGTTCTGATCACCGTACTC. |
| ABCG2 | Forward primer (5'-3'), CAGGTGGAGGCAAATCTTCGT; |
| | Reverse primer (3'-5'), ACCCTGTTAATCCGTTCGTTTT. |

**Supplementary Table S3. The antibodies used in this study**

| Antibodies | Catalog# | Source |
|---|---|---|
| anti-METTL3 | ab195352 | Abcam |
| anti-ABCC2 | ab172630 | Abcam |
| anti-$m^6A$ | ab151230 | Abcam |
| anti-Ki67 | 550609 | BD Pharmingen |
| anti-YTHDF1 | R27444 | zenbio |
| anti-cyclin B1 | R23304 | zenbio |
| anti- Goat Anti-Rabbit IgG H&L (AF488) | 550037 | zenbio |

| | | |
|---|---|---|
| anti-γH2AX (phospho S139) | 3924583 | Merck |
| anti-BCL-2 | 12789-1-AP | proteintech |
| anti-β-Actin | sc-1616 | Santa Cruz |
| anti-pan-cadherin | R25274 | zenbio |
| anti-BAX | 380709 | zenbio |
| anti-caspase 3 | sc-56053 | Santa Cruz |

**Supplementary Table S4. The primers used for MeRIP-qPCR**

| Site | **Primer sequences** |
|---|---|
| ABCC2-m$^6$A | Forward primer (5’-3’), TGAAGGAAGACGAAGAAC;<br>Reverse primer (3’-5’), CTTCACCTTTCCAGTTTCTATG. |
